# Contribution of Orb2A Stability in Regulated Amyloid-Like Oligomerization of *Drosophila* Orb2

**DOI:** 10.1371/journal.pbio.1001786

**Published:** 2014-02-11

**Authors:** Erica White-Grindley, Liying Li, Repon Mohammad Khan, Fengzhen Ren, Anita Saraf, Laurence Florens, Kausik Si

**Affiliations:** 1 Stowers Institute for Medical Research, Kansas City, Missouri, United States of America; 2 Department of Molecular and Integrative Physiology, University of Kansas Medical Center, Kansas City, Kansas, United States of America; Baylor College of Medicine, United States of America

## Abstract

How learned experiences persist as memory for a long time is an important question. In *Drosophila* the persistence of memory is dependent upon amyloid-like oligomers of the Orb2 protein. However, it is not clear how the conversion of Orb2 to the amyloid-like oligomeric state is regulated. The Orb2 has two protein isoforms, and the rare Orb2A isoform is critical for oligomerization of the ubiquitous Orb2B isoform. Here, we report the discovery of a protein network comprised of protein phosphatase 2A (PP2A), Transducer of Erb-B2 (Tob), and Lim Kinase (LimK) that controls the abundance of Orb2A. PP2A maintains Orb2A in an unphosphorylated and unstable state, whereas Tob-LimK phosphorylates and stabilizes Orb2A. Mutation of LimK abolishes activity-dependent Orb2 oligomerization in the adult brain. Moreover, Tob-Orb2 association is modulated by neuronal activity and Tob activity in the mushroom body is required for stable memory formation. These observations suggest that the interplay between PP2A and Tob-LimK activity may dynamically regulate Orb2 amyloid-like oligomer formation and the stabilization of memories.

## Author Summary

The formation of stable long-term memories involves the synthesis of new protein, however the biochemical basis of this process is unclear. A family of RNA binding proteins, Cytoplasmic Polyadenylation Element Binding (CPEB) proteins, are known to regulate synaptic activity and stabilization of memory. The *Drosophila* CPEB is called Orb2, and its amyloid-like oligomers are critical for the persistence of long-lasting memories. Amyloid formation is often unregulated and stochastic in nature, and the amyloid state is usually dominant and self-sustaining. However, to serve as a substrate for long-lasting memory, the amyloid-like oligomerization of Orb2 must be regulated in a space-, time-, and stimulus-specific manner. Orb2 has two protein isoforms: Orb2A, which is present only in low abundance, and Orb2B, which is the abundant form. Orb2A is important for oligomerization as well as memory persistence. Previous studies suggested that Orb2A may act as a seed to induce oligomerization of the constitutive Orb2B isoform. Therefore, the availability of Orb2A protein would be an important determinant of Orb2 oligomerization. Here we have analyzed how Orb2 conversion to the oligomeric state is regulated. We find that Orb2A is a very unstable protein and that phosphorylation-dephosphorylation of this isoform via canonical neuronal signaling modules can regulate Orb2A stability, and thereby its abundance. We also show that Tob, a known regulator of CPEB-mediated translation, acts as a stabilizer for Orb2A and triggers Orb2 oligomerization. These observations suggest that amyloid formation can be regulated in a dynamic manner by controlling the availability of the seeding Orb2A protein.

## Introduction

Synthesis of new protein is important for the formation of stable memory [1]. The Cytoplasmic Polyadenylation Element Binding (CPEB) proteins are a family of RNA binding proteins that regulate the translation and subcellular distribution of a specific set of cellular mRNAs in various cell types including neurons [2]. Previous studies found that some CPEB family members play a causal role in long-term change of synaptic activity and in stabilization of memory [3]–[9]. For example, in marine snail *Aplysia*, in the absence of a neuron-specific *Ap*CPEB, serotonin mediated enhancement of synaptic transmission fails to persist beyond 24 h [7],[10]. Likewise, the *Drosophila* CPEB, Orb2, is required specifically for long-term memory but not for learning or short-term memory [3]–[5]. In humans, a particular CPEB family member, CPEB3, has been linked to episodic memory formation, suggesting a conserved role of CPEB in synaptic plasticity and memory [11].

Interestingly, *Ap*CPEB and Orb2 form self-sustaining amyloidogenic oligomers (prion-like) in response to the neurotransmitters serotonin in *Aplysia* and octopamine or tyramine in *Drosophila* [5],[6],[12],[13]. More importantly, the oligomeric CPEB is required for the persistence of synaptic facilitation in *Aplysia* [6] and for the stabilization of memory in *Drosophila* [5]. These observations led us to propose that the persistent form of memory recruits an amyloidogenic oligomeric form of neuronal CPEB to the activated synapse, which in turn maintains memory through the sustained, regulated synthesis of a specific set of synaptic proteins [5]. However, considering the dominant and stable nature of amyloids, a central question is how the conversion of neuronal CPEB to the amyloidogenic state is regulated to confer activity dependence and restrict it to the relevant neuron/synapse.

The *Drosophila* Orb2 gene has two protein isoforms, Orb2A and Orb2B, and the oligomers are composed of both Orb2A and Orb2B. In the adult brain, in comparison to the Orb2B protein, the Orb2A protein is expressed at an extremely low level [4],[5]. In spite of its low abundance, the Orb2A protein is critical for Orb2 oligomerization, and Orb2A forms oligomers more readily than Orb2B. More importantly, a mutation that impedes Orb2A oligomerization selectively affects persistence of memory [5], and the Orb2A prion-like domain is sufficient for long-term memory formation [4]. These observations suggested a model in which the rare Orb2A protein either acts directly as a seed to induce activity-dependent amyloid-like oligomerization of the constitutive Orb2B protein or Orb2A oligomerization indirectly affects oligomerization of Orb2B [5]. In either case the amount and localization of Orb2A protein would therefore be a key determinant of when and where amyloid-like conversion would occur.

Here we find that Orb2A has a very short half-life and the Orb2 interacting protein Transducer of Erb2 (Tob), a known regulator of cellular growth, stabilizes Orb2A and induces Orb2 oligomerization. Expression of dsRNA against Tob in the mushroom body neurons does not affect learning, but impairs long-term memory formation. Tob recruits the neuronal protein kinase Lim Kinase (LimK) to the Tob-Orb2 complex to induce Orb2 phosphorylation. Phosphorylation regulates Tob-Orb2 association as well as the stability of both proteins, and Protein Phosphatase 2A (PP2A) is a key regulator of the phosphorylation status of Tob and Orb2. Intriguingly, inhibition of PP2A stabilizes Orb2A, but destabilizes Orb2-associated Tob, providing a mechanism for temporal restriction on Orb2A stabilization. Since PP2A and LimK activity can be regulated in a synapse-specific manner [14],[15], the phosphorylation-dephosphorylation of Orb2 and Tob provides a putative mechanism of restricting the Orb2 oligomerization to the activated synapse. Tob is also known to regulate the function of CPEB family members [16]. Therefore, the Tob-Orb2 association-dissociation may also regulate Orb2 function in the nervous system.

## Results

### Orb2 Interacting Proteins in the Adult *Drosophila* Brain

**Figure 1:**
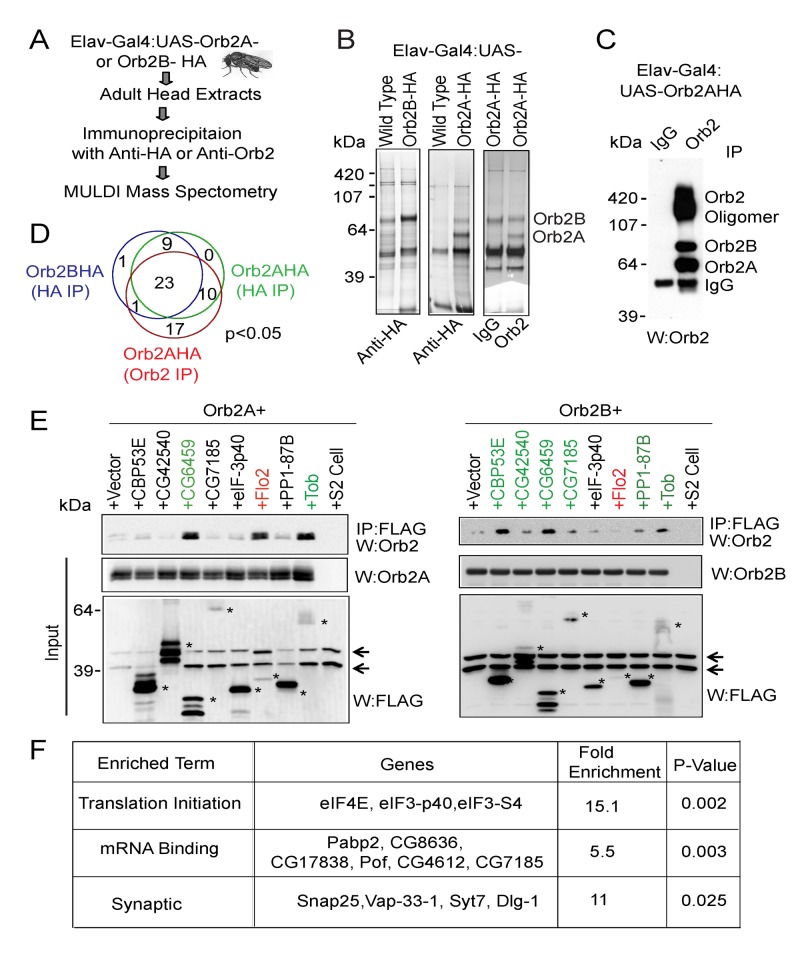
Identification of Orb2-interacting proteins from the adult *Drosophila* brain. (A) Schematic of the experimental design. (B) Representative examples of silver-stained gels of anti-HA IP of Orb2BHA (left panel) or Orb2AHA (middle panel) and anti-Orb2 IP of Orb2AHA (right panel) used for proteomic analysis. The overexpressed monomeric Orb2A and Orb2B proteins are visible in the silver-stained gels. The wild-type flies serve as a control for HA transgene, and purified guinea pig IgG serves as a control for anti-Orb2 antibody. (C) Anti-Orb2 antibody immunopurified both monomeric and amyloid-like oligomeric Orb2 from Orb2AHA expressing fly head extracts. (D) The distribution of 61 proteins that were significantly enriched in the Orb2 immunoprecipitates over control. (E) Representative IP–Western blots of candidate proteins that were tested for pair-wise interaction with Orb2 in S2 cells. The FLAG-tagged putative candidate proteins were coexpressed with untagged Orb2 proteins, immunoprecipitated with anti-FLAG antibodies, and Western blotted with anti-Orb2 antibody. The proteins indicated in green have consistently shown above background binding. The flotilin (Flo2) gene, indicated in red, was overrepresented but not significantly enriched in Orb2 immunoprecipitate. However, it binds to Orb2A. The asterisk indicates the position of candidate proteins in SDS-PAGE. The arrows indicate anti-FLAG antibody cross-reacting polypeptides in S2 cell extracts. Unless indicated otherwise, in IP experiments 5% of the lysate is used as loading controls. (F). Gene Ontology (GO) enrichment analysis of Orb2 proteome. The FBgn IDs of the candidate Orb2-interactors were submitted to DAVID web server. Selected nonredundant terms are shown. The *p* values and fold enrichments were determined using the *Drosophila* melanogaster genome as background. Uncorrected *p* values are shown due to prior filtering for enriched peptides and the small input sample size. Also see Figure S1 and Table S1.

A regulator of Orb2 oligomerization could potentially fall into at least two distinct categories: an activator that associates with Orb2 and facilitates conversion to the oligomeric state or a repressor that binds to Orb2 and prevents its oligomerization. To identify both types of regulators we used a proteomics approach to perform a comprehensive search for Orb2 interacting proteins in the adult *Drosophila* brain. The Orb2 proteins were expressed pan-neuronally as C-terminal HA-tagged proteins (ElavGal4: UAS-Orb2AHA or Orb2BHA), and the Orb2 complex was immunopurified using anti-HA antibodies from RNaseA-treated adult head extract (Figure 1A and B). Previously we observed that the C-terminal tags are inaccessible in the Orb2 oligomeric state [5]; thus, the anti-HA antibody preferentially immunopurified the Orb2 monomers. Therefore, to identify proteins that interact with oligomeric Orb2, we also immunopurified Orb2AHA with an anti-Orb2 antibody (Figure 1B and C). The factors associated with Orb2 were identified using Multidimensional Protein Identification technology (MudPIT) (Table S1) [17].

We found 61 proteins that were significantly enriched (*p*<0.05) in the Orb2 immunoprecipitates compared to eight independent control immunoprecipitates (Figures 1D and S1A and Table S1). Eleven proteins were overrepresented in Orb2 IP compared to the controls, albeit not to statistical significance (Table S1). To determine the validity of the proteomic approach, we randomly sampled 20 candidate proteins (out of 72 proteins) by pair-wise interaction in S2 cells (Figure 1E and Figure S1B). Approximately 50% (11 out of 20 proteins) of the proteins thus tested formed a complex with at least one of the Orb2 proteins in an RNA-independent manner (Figure 1E and Figure S1B). Therefore, the proteomics approach indeed identified specific components of an Orb2 protein complex in the adult *Drosophila* brain. The candidate proteins either interact directly with Orb2 or indirectly as part of a larger Orb2 protein complex. A gene ontology (GO) analysis revealed that the Orb2 proteome is significantly enriched for proteins involved in translation initiation, mRNA binding, and synaptic activity (Figure 1F). The enrichment of these protein complexes supports the idea that Orb2 is involved in regulation of synaptic protein synthesis.

### *Drosophila* Tob Stabilizes Orb2A

**Figure 2:**
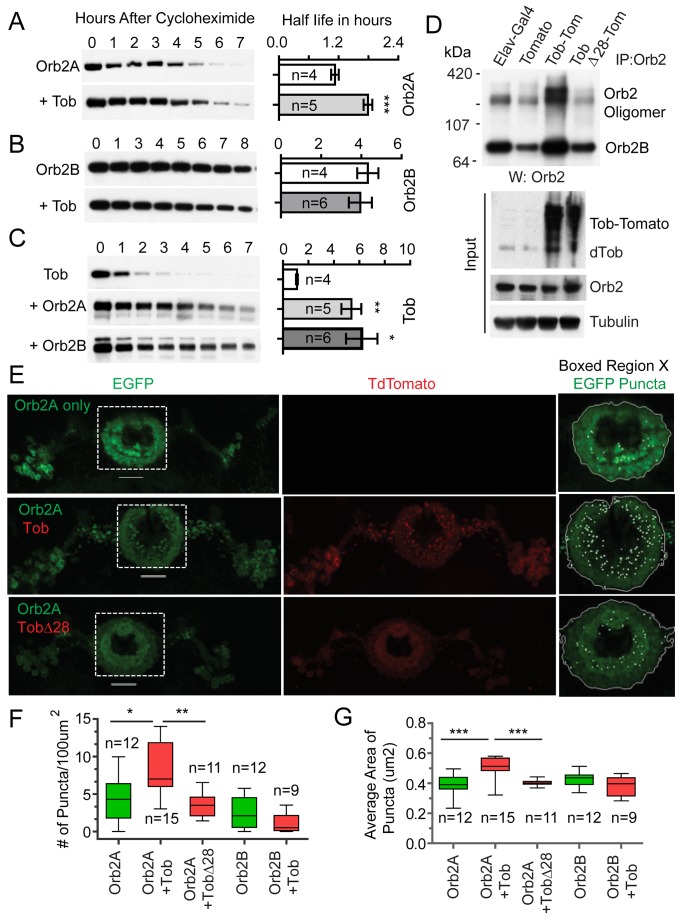
Tob stabilizes Orb2A and induces Orb2 oligomerization. (A) Tob enhances Orb2A stability. Orb2 stability was examined in S2 cells expressing either Orb2A by itself (top panel, Orb2A) or in conjunction with Tob (bottom panel, +Tob). Following the addition of cycloheximide (Chx), samples were taken at the given time points and then analyzed for expression levels of Orb2A by Western blot (left panels). Right panels compare protein half-lives. Half-lives were determined by plotting the percent of protein remaining after time zero and assuming first-order kinetics. The *n* indicates number of independent experiments performed to determine half-lives. Statistical significance was determined using an unpaired, two-tailed *t* test. The data are plotted as mean ± SEM. (B) Tob has little effect on Orb2B stability. (C) Both Orb2A and Orb2B significantly enhance Tob stability. (D) Overexpression of Tob increases Orb2 oligomerization. Orb2 was immunoprecipitated from adult head extracts expressing only TdTomato (Elav-Gal4: UAS-TdTom), Tob tagged to TdTomato (Elav-Gal4: UAS-TobTdTom), or Tob lacking a 28 amino acid domain critical for binding to Orb2 (Elav-Gal4: UAS-TobΔ28TdTom). (E) Overexpression of Tob increases the number and size of Orb2AEGFP puncta. (Left panel) Both proteins were expressed in the ellipsoid body of the central complex using a c547Gal4 driver. Each row represents a fly genotype: c547-Gal4:UAS-Orb2AEGFP (Orb2A only), c547-Gal4:UAS-Orb2AEGFP/UAS-TobTdtomato (Orb2A Tob), and c547-Gal4:UAS-Orb2AEGFP/UAS-TobΔ28Tdtomato (Orb2A TobΔ28). Scale bar, 25 µm. (Right panel) Higher magnification image of the boxed region in the left. Puncta were counted in the central portion of the ellipsoid body. Axiovision software was programmed to identify a continuous central region and define aggregates, indicated as white dots. Scale bar, 20 µm. Please see Figure S3 for additional images. (F) Puncta number (/100 µm^2^) and (G) size (average area of aggregates in µm^2^) were quantified. Statistical analysis was performed using an unpaired two-tailed *t* test (*) *p*≤0.05, (**) *p*≤0.01, and (***) *p*≤0.001. *n*, the number of flies examined for each genotype. The data are plotted as mean ± SEM. Also see Figures S2 and S3.

The Orb2A protein is undetectable by Western analysis, and a genomic construct expressing Orb2A-EGFP suggests it is ∼100 times less abundant than Orb2B protein in the adult brain [5]. Moreover, monomeric Orb2A has a very short half-life compared to Orb2B (Figure 2A and Table S2). Taken together, these observations suggest that availability of the Orb2A protein could be an important determinant of efficient Orb2A oligomerization and/or function. In the course of our interaction studies in S2 cells, we noticed one of the candidate proteins, Tob, may influence the Orb2A protein level (Figure 1E). To determine Orb2A and Orb2B stability independent of each other, we used *Drosophila* S2 cells, in which Orb2 is normally not expressed and Tob is expressed at low levels. S2 cells were transfected with only HA-tagged Orb2 or coexpressed with Flag-tagged Tob. To determine half-life, total Orb2 or Tob protein levels were measured at several time points following treatment with cycloheximide (CHX), which blocks new protein synthesis. The coexpression of Tob nearly doubled the half-life of monomeric Orb2A (Figure 2A). However, Tob had no significant effect on Orb2B (Figure 2B), indicating that association with Tob does not automatically enhance half-life. Likewise, incubation with dsRNA against Tob reduced the level of Orb2A protein but not Orb2B (Figure S2A). Earlier studies with Tob family members have suggested that the stability of Tob itself can be regulated [18],[19]. We found a fourfold increase in Tob half-life in the presence of either Orb2A or Orb2B compared to Tob alone (Figure 2C and Table S2). These results suggest that not only does Tob stabilize Orb2A, but Orb2 proteins have stabilizing effects on Tob.

The recombinant *Drosophila* Tob interacts with *in vitro* transcribed and translated Orb2 proteins, suggesting direct interaction between these proteins (Figure S2B). In mammals, the Tob family consists of six members, with *Drosophila* Tob most closely related to the mammalian Tob1 and Tob2 proteins [20]. We found both *Aplysia* CPEB and mouse CPEB3 interact with the closely related Tob1 and Tob2, and Tob2 increases the steady-state level of ApCPEB and CPEB3 (Figure S2C). Recently, others have reported a direct interaction between mouse CPEB3 and Tob1 [16], suggesting that Tob is an evolutionarily conserved interactor of CPEB proteins. Tob is required for long-term potentiation of hippocampal CA1–CA3 synapses, a cellular correlate of long-term memory in mammals [21], and Tob activity is modulated by bone morphogenetic proteins or BMPs [22]–[24]. These observations suggest Tob could function as an extracellular signal-dependent regulator of Orb2 in the nervous system.

### Tob Enhances Orb2 Amyloid-Like Oligomerization in the *Drosophila* Brain

Does Tob influence Orb2 oligomerization in the adult fly brain? To answer this, we increased Tob level in the fly brain using the Gal4-UAS system and assessed Orb2 oligomerization by immunopurification. Overexpression of Tob-TdTomato (Elav-Gal4: UAS-TobTdTom), but not the fluorophore alone (Elav-Gal4: UAS-TdTom), increased the levels of 10% SDS and boiling-resistant oligomeric Orb2 in the fly brain (Figure 2D). The amount of Orb2 oligomers in Tob-expressing flies increased nearly 2-fold compared to control flies (fold increase in oligomers normalized to monomer ± SEM, 1.95±0.27, *p*<0.05, *t* test). Tob has been implicated in a number of cellular processes, including transcriptional regulation and RNA metabolism [23],[25]–[28], raising the possibility that the increase in Orb2 oligomerization is a secondary effect of Tob overexpression. We generated a series of deletion mutants of Tob and found that deletion of a conserved 28 amino acid motif, TobΔ28 (Figure S3A), decreased the interaction between Tob and Orb2 in both S2 cells (Figure S3B) and the adult fly brain (Figure S3C). However, it had no effect on the association between Tob and the deadenylase Pop2 (Figure S3D) or with the transcriptional repressor, Mad (Figure S3E). Overexpression of TobΔ28 (Elav-Gal4: UAS-TobΔ28TdTom) in the adult brain did not enhance Orb2 oligomerization (fold increase in oligomers normalized to monomer ± SEM, 0.5±0.14) (Figure 2D).

Does Tob enhance oligomerization of Orb2A, Orb2B, or both? EGFP-tagged Orb2A and Orb2B formed stable oligomers in the adult fly brain and the oligomers associated with Tob (Figure S3F). To determine the effect of Tob on Orb2A and Orb2B, we coexpressed TdTomato-tagged Tob with EGFP-tagged Orb2A or Orb2B in the adult fly brain. To distinguish from the endogenous Orb2, we quantified changes in the number of fluorescent puncta, since the abundance of fluorescence puncta co-relate with extent of oligomerization (Figure 2E) [5]. The number of Orb2A-EGFP puncta increased ∼2-fold in the presence of Tob (number of puncta/100 µm^2^ ± SEM, Orb2A: 4.41±2.88, *N* = 12; Orb2A+Tob: 8.06±3.69, *N* = 15, *t* test, *p* = 0.012) but not in the presence of TobΔ28 (3.58±1.62, *N* = 11, *t* test, *p*>0.5) (Figure 2F). Unlike Orb2A, Orb2B∶EGFP by itself remained mostly diffused and Tob overexpression had no significant effect on the rare Orb2B puncta (number of puncta/100 µ∶m^2^ ± SEM: Orb2B, 2.40±2.11, *N* = 12, Orb2B+Tob, 1.07±1.30, *N* = 9, *t* test, *p* = 0.155) (Figure 2F and Figure S3G). In addition to being more numerous, the size of Orb2A puncta also increased significantly when Tob was overexpressed (size of puncta ± SEM; Orb2A, 0.39±0.08 µm^2^, Orb2A+Tob, 0.51±0.07 µm^2^, *p* = 0.0003), an effect not seen with the rare Orb2B puncta (Orb2B, 0.43±0.05 µm^2^, Orb2B+Tob, 0.38±0.06 µm^2^, *p* = 0.20) (Figure 2G). Taken together, these observations suggest that Tob-Orb2 association promotes Orb2 oligomer formation either by increasing the Orb2A protein levels and/or enhancing oligomerization.

### Neuronal Stimulation Enhances Tob-Orb2 Association

**Figure 3:**
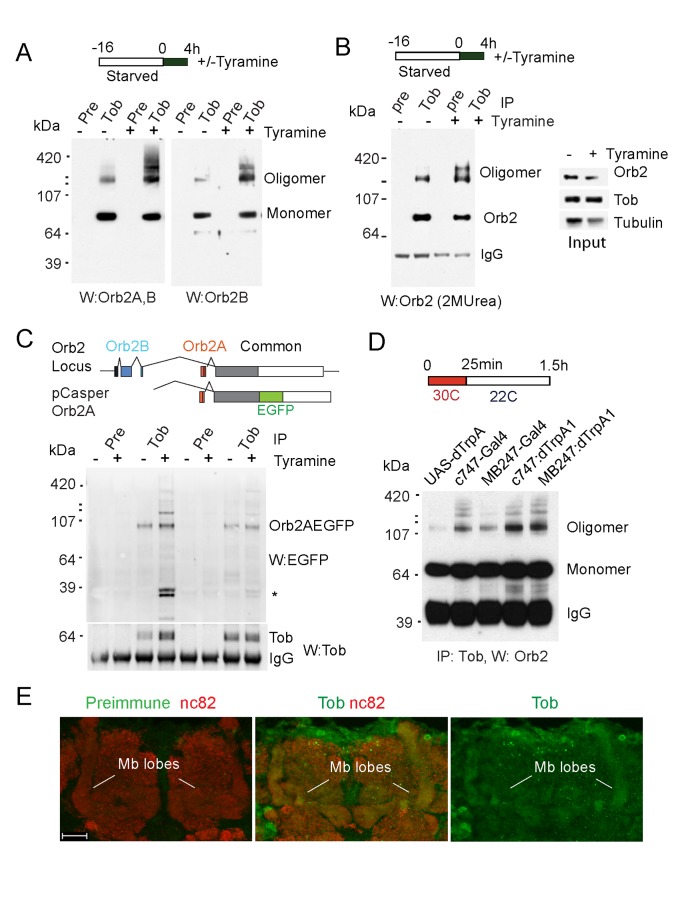
Tob-Orb2 association is regulated by neuronal activity, and Tob is present in the same synaptic compartment as Orb2. (A) Neuronal stimulation increases Tob-Orb2 association. Tob was immunoprecipitated from unstimulated (control) or 10 mM tyramine-stimulated head extracts and blotted with the anti-Orb2 antibody (Orb2A,B) that detects both forms of Orb2 or an antibody that detects only Orb2B (Orb2B). The preimmune serum (pre) from the same animal serves as control for Tob antibody specificity. (B) Tob-associated oligomeric Orb2 is amyloidogenic. Tob was immunoprecipitated from either unstimulated (control) or tyramine-stimulated head extracts, treated with 10% SDS and 2 M urea and blotted with the anti-Orb2 antibody. Western analysis of lysates indicates the expression levels of Orb2, Tob, and tubulin. (C) Tyramine increases Tob-Orb2A association. (Top panel) A schematic diagram of the genomic pCasperOrb2A construct, which expresses an EGFP-tagged Orb2A. The Orb2B-specific exons are indicated in blue, Orb2A-specific exon in red, and the common region in gray. (Bottom panel) Tob was immunoprecipitated from unstimulated (−) or tyramine-stimulated (+) total brain lysates with preimmune (pre) or immune serum (Tob) and probed with anti-EGFP antibody. The position of the monomeric Orb2AEGFP fusion protein is indicated. The EGFP-antibody reacting high molecular weight proteins are most likely the oligomeric form of the Orb2AEGFP protein. The asterisk indicates low molecular weight protein that is either a degradation product of Orb2AEGFP or a cross-reactive band. In the lower panel Tob is visible only in immune serum lane, indicating specificity of the pull-down. Orb2AEGFP is not detectable by Western analysis. (D) Stimulation of mushroom body neurons enhances Tob-Orb2 association. A schematic of the stimulation protocol is shown above the gel picture. The mushroom body neurons were stimulated using the temperature-sensitive dTrpA1 channel, which depolarizes and thereby activates neurons at a temperature >25°C. More Tob-associated Orb2 oligomers were observed at 30°C in flies harboring both the Gal4 driver and TrpA1 transgene compared to flies with just the Gal4-driver or TrpA1. The c747-Gal4 and MB247-Gal4 drive expression in all neurons of the adult *Drosophila* mushroom body. (E) Tob is widely distributed in the adult brain. Frontal cryosections of adult fly heads were immunostained with the preimmune serum or anti-Tob (green) antibody. Tob is present in the cell body and at a low level in the synaptic neuropil region of mushroom body (Mb lobes) Kenyon cells. Nc82 (red) marks the synaptic neuropil region. Scale bar, 20 µm. Also see Figure S4.

Is Tob involved in activity-dependent oligomerization of Orb2? Previously we and others have observed that a neurotransmitter such as tyramine or dopamine regulates Orb2 oligomerization [4],[5]. Therefore, we checked whether tyramine modulates Orb2-Tob interaction. To this end, we fed-starved flies 10 mM tyramine and after 4 h immunopurified the Tob-Orb2 complex from tyramine-stimulated or -unstimulated adult fly brain using a *Drosophila* Tob-specific antibody (Figure S4A). Tyramine stimulation increased the Tob-bound oligomeric Orb2 nearly 4-fold (fold increase in oligomers normalized to monomer ± SEM, 3.82±0.88, *n* = 5, *t* test, *p*<0.05) (Figure 3A), and the oligomers are resistant to boiling in the presence of 10% SDS and 2 M urea, consistent with it being amyloid-like (Figure 3B). The neurotransmitter serotonin (5-HT) had less effect on Tob-Orb2 association (Figure S4B), consistent with our earlier observation that Orb2 oligomerization is influenced by tyramine and not by 5-HT [5]. Use of Orb2B-specific antibody (Figure 3A, right panel) indicated Tob-Orb2B association is enhanced by tyramine stimulation. To determine whether Tob-Orb2A association is also modulated by neuronal activity, we used a genomic construct that encompasses only Orb2A coding region and carries EGFP at the C-terminal end (pCasperOrb2AEGFP) [5]. In Tob immunoprecipitate from tyramine-treated samples, we see EGFP reacting bands that correspond to the size of the monomeric- (∼87 KDa) and oligomeric-Orb2AEGFP (Figure 3C). Since it is difficult to determine which neuronal populations are activated by tyramine feeding, we also directly activated the mushroom body neurons (c747-Gal4, MB247-Gal4) with the temperature-sensitive dTrpA1 channel [29]. The flies were transiently exposed to 30°C (dTrpA1 active) for 25 min and then returned to 22°C (dTrpA1 inactive). Compared to flies carrying only dTrpA1 or Gal4, flies carrying both transgenes (C747Gal4::UAS-dTrpA1 or MB247Gal4:UAS-dTrpA1), there was enhanced Tob-Orb2 association (Figure 3D). Taken together these observations suggest that neuronal activity that enhances Orb2 oligomerization also enhances Tob-Orb2 association.

Because Tob was initially identified as a transcriptional regulator [23],[24], we asked whether Tob is restricted to the cell body or distributed throughout the neuron, including the synaptic region. Immunostaining of the adult fly brain revealed that, as expected, Tob is present mostly in the cell body (Figure S4C). However, at low levels Tob staining was also detected in the synaptic-neuropil regions (Figure 3E, mushroom body lobes). Previously we established a method to purify synaptosomes from adult *Drosophila* head [5]. In Western blotting of synaptosome fractions (Figure S4D, left panel) Tob was found in the synaptic membrane fraction, similar to Orb2 (Figure S4D). In Δ80QOrb2 flies, which has significantly less Orb2 protein compared to wild-type flies [5], the distribution of Tob was unaffected, suggesting synaptic localization of Tob is independent of Orb2 (Figure S4D). Similar to the fly brain, Tob was also detected in the synaptic membrane fraction prepared from the mouse brain (Figure S4E). Activity-dependent association with Orb2 and presence in the synaptic region suggest that Tob may act to regulate Orb2 function and/or oligomerization in the synapse.

### Phosphorylation Regulates Tob-Orb2 Association

**Figure 4:**
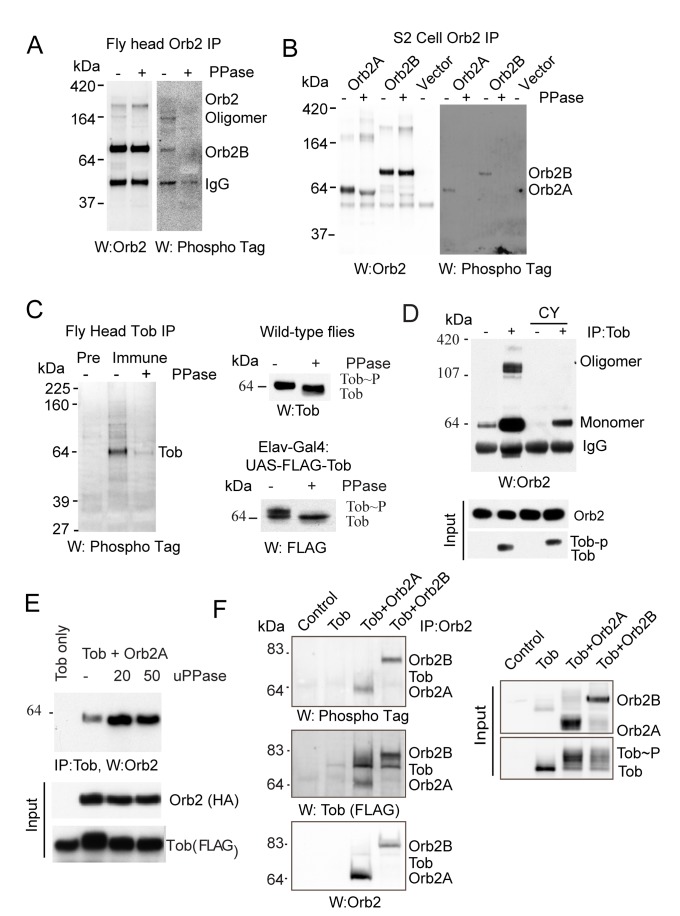
Tob and Orb2 are phosphorylated, and phosphorylation regulates Tob-Orb2 association. (A) Orb2 is phosphorylated in the adult fly head. Blotting of Orb2 immunoprecipitate with phospho-tag™ detects phosphorylated Orb2B. Treatment with calf-intestinal phosphatase (PPase) that removes phosphate groups from proteins shows specificity of phospho-tag™. (B) Both Orb2A and Orb2B are phosphorylated at a low level in S2cells. Orb2 immunoprecipitate from the S2 cell is probed with phospho-tag™. (C) Tob is phosphorylated in the adult fly brain. (Left panel) Approximately 2 mg of total head extracts were immunoprecipitated with preimmune (pre) or immune Tob serum. The phospho-tag™ detects a band at the position of Tob only in the immune but not in preimmune lane. Treatment with λ-phosphatase (PPase) reduces phospho- tag™ signal. (Right panel, top) Total adult head extracts were treated with calf-intestinal phosphatase (PPase). Change in phosphorylation status of Tob was assessed as a change in mobility by Western blot analysis. (Right panel, bottom) Exogenously expressed Flag-tagged Tob protein is also phosphorylated in the adult brain. (D) The addition of the phosphatase inhibitor, calyculin (CY), dissociates the Orb2-Tob complex. S2 cells were transfected with Orb2A with and without Tob and treated with 10 µm CY for 1 h prior to Tob immunoprecipitation. CY treatment almost completely abolished Tob association with Orb2A oligomers and reduced association with the monomers. (E) Unphosphorylated Tob has a greater affinity for Orb2A. S2 cell lysates were first treated with the indicated units of phosphatase (uPPase), and subsequently the Orb2A-Tob complex was immunoprecipitated. More Orb2A was found to be associated with Tob following phosphatase treatment. Western blots of the lysates show the level of Orb2A and Tob (input). The 4%–12% gradient gels were used in these experiments. (F) Hyperphosphorylated Tob does not associate with Orb2. Untagged Orb2 and FLAG-tagged Tob complex was immunopurified using anti-Orb2 antibodies and probed with phosphor-tag™ (top panel). Phosphorylated proteins correspond to the size of Orb2 (bottom panel) but not Tob (middle panel). The hyperphosphorylated Tob proteins are visible in the total extract (input), however they are absent in the Orb2-Tob complex. The 8% gel in Tris-Glycine buffer was used in this experiment.

Because Tob is constitutively present in the adult fly brain, we wondered how Tob-mediated oligomerization of Orb2 could be temporally regulated by neuronal activity. Phosphorylation is known to regulate the activity of both Btg/Tob [30]–[32] as well as the CPEB family members [33]–[35]. Consistent with these observations, protein phosphatase 1 (PP1-87B) and protein phosphatase 2A (PP2A) regulatory subunit twins were found in the Orb2 protein complex (Table S1), suggesting that Orb2 may also be regulated via phosphorylation and/or that Orb2 recruits these phosphatases to regulate phosphorylation of other proteins (such as Tob) in the complex. Blotting of Orb2 immunoprecipitates from the adult brain with phospho-tag™ [36], a biotin-tagged dinuclear metal complex that selectively binds to phospho-proteins, detected a small amount of phosphorylated monomeric Orb2B protein (Figure 4A). Similar to the fly brain, when expressed ectopically in S2 cells, both Orb2A and Orb2B are phosphorylated, albeit at very low levels (Figure 4B), suggesting Orb2 proteins are transiently phosphorylated in a regulated manner or kept primarily in an unphosphorylated state by the phosphatase. We observed that Tob is also phosphorylated in the adult fly brain (Figure 4C). To avoid a secondary consequence of prolonged inhibition or activation of phosphatases or kinases in the nervous system, we took advantage of the phosphorylation of Orb2 and Tob in S2 cells to determine the acute role of phosphorylation.

To determine if phosphorylation has any effect on Tob-Orb2 association, we blocked dephosphorylation using calyculin (CY), a cell-permeable serine-phosphatase inhibitor that blocks protein phosphatase 2A (PP2A) at 0.5–1.0 nM concentration and protein phosphatase1 (PP1) at ≥2 nM concentration [37]. We observed that an hour after treatment with 1 nM CY, the amount of Orb2A associated with Tob was reduced (Figure 4D). The reduction in association was not due to reduction in Tob or Orb2A protein level an hour after treatment with CY (Figure 4D). Because phosphatases influence a large number of proteins in the cell, the reduction of Tob-Orb2 association could be a secondary consequence of phosphatase inhibition. To test more directly the effect of phosphorylation, in a reciprocal experiment, we first treated cell lysates expressing Tob and Orb2A with calf intestinal phosphatase (CIP) and then isolated the Tob-Orb2 complex (Figure 4E). We observed that prior dephosphorylation enhanced the association of Tob with Orb2A (Figure 4E). Likewise, when the Orb2-Tob complex was immunopurified with anti-Orb2 antibody and probed with phospho-tag™, only phosphorylated Orb2, but not the hyperphosphorylated Tob, was detected in the immunoprecipitate (Figure 4F). Taken together, these results indicate phosphorylation regulates Tob-Orb2 association. Hypophosphorylation promotes Tob-Orb2A association, and hyperphosphorylation reduces it.

### PP2A Inactivation Destabilizes Tob But Stabilizes Orb2A

**Figure 5:**
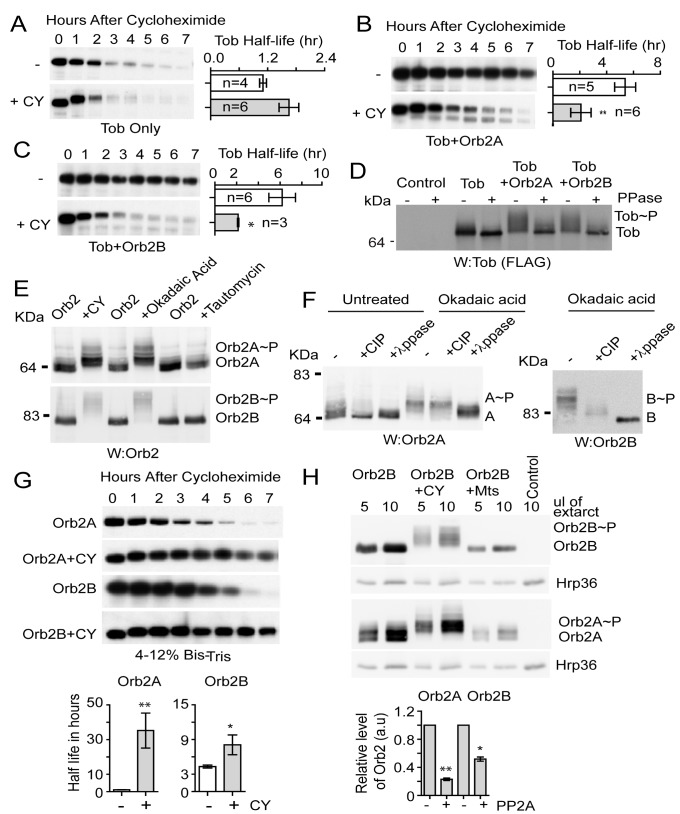
PP2A regulates Tob-Orb2 phosphorylation and stability. (A–C) Tob is destabilized upon PP2A inhibition when in complex with Orb2. (A) The phosphatase inhibitor calyculinA (CY) does not significantly affect the half-life of Tob alone. (B and C) In the presence of CY, the increase in Tob stability normally observed in the presence of Orb2A (B) or Orb2B (C) was significantly reduced. (D) In S2 cells Tob is phosphorylated when coexpressed with Orb2A or Orb2B. Changes in Tob phosphorylation was confirmed by λ-phosphatase (PPase) treatment. (E) PP2A inhibitor CY, okadaic acid, but not PP1 inhibitor tautomycin increases Orb2A and Orb2B phosphorylation, as evident in shift electrophoretic mobility. (F) The Orb2 proteins are phosphorylated in multiple sites. (G) PP2A inhibitor CY increases the half-life of Orb2A and Orb2B. (H) Overexpression of PP2A catalytic subunit Mts destabilizes Orb2A and Orb2B. The RNA binding protein Hrp36 serves as loading control. Statistical significance was measured with two-tailed *t* test (*) *p*≤0.05, (***) *p*≤0.001. *n*, the number of independent experiments for each experimental group. The shift in molecular weight associated with phosphorylation was assayed in 8% SDS-PAGE. The half-life determination experiments were assayed in 4%–12% SDS-PAGE. Also see Figure S5.

Because the Tob-Orb2 association alters the half-life of both proteins and phosphorylation affects their association, we examined the effect of phosphatase inhibition on the half-life of both proteins. When Tob was expressed by itself there was modest change in stability in the presence of CY (Table S2) compared to the untreated samples (Figure 5A). Interestingly, the increase in Tob stability that occurred when co-expressed with either Orb2A (Figure 5B) or Orb2B (Figure 5C) was ∼50% reduced when the phosphatases were inhibited (Table S2). The destabilization of Tob was observed only in the presence of the PP2A/PP1 inhibitor CY or okadaic acid (1 nM) but not the PP1 selective inhibitor tautomycin (10 nM) (Figure S5A) [37],[38]. Moreover, the extent of Tob phosphorylation appears to be specifically linked to Orb2 complex formation (Figure 5D). The Orb2 proteins, but not the other homologue of CPEB in *Drosophila*, Orb1, enhance phosphorylation of Tob, although Tob interacts with both Orb2 and Orb1 (Figure S5B and C). These results suggest un- or hypophosphorylated Tob binds Orb2. Association of Tob with Orb2 and PP2A inactivation leads to additional phosphorylation of Tob-Orb2, which results in dissociation and eventual destabilization of Tob.

How does phosphorylation affect Orb2? Treatment of S2 cells with PP2A/PP1 inhibitors CY (1 nM) and okadaic acid but not PP1-specific inhibitor tautomycin (10 nM) enhanced phosphorylation of both Orb2A and Orb2B (Figure 5E and Figure S5D). Treatment with alkaline phosphatase, which removes phosphate from serine/threonine, and λ phosphatase, which removes phosphate from serine/threonine as well as tyrosine residues [39], revealed that upon inhibition of PP2A, Orb2 proteins are phosphorylated at multiple sites (Figure 5F). One of the outcomes of these multiple phosphorylations is a significant increase in Orb2A half-life, from 1 h to >24 h, *t*(1/2) Orb2A, 1.13±0.08, Orb2A+CY, 35.5±17.5 h; *p* = 0.010, and doubling of the Orb2B half-life, *t*(1/2) Orb2B, 4.32±0.53, Orb2B+CY, 8.09±2.95 h, *p* = 0.05 (Figure 5G). As decreases in PP2A activity increased Orb2 level, likewise increases in PP2A activity by overexpression of PP2A catalytic subunit microtubule star (Mts) that associates with Orb2 (Figure S5E) resulted in a ∼4-fold decrease in Orb2A (0.23±0.01, *n* = 5) and a ∼2-fold decrease in Orb2B (0.51±0.02, *n* = 3) protein level (Figure 5H). Increases or decreases in protein phosphatase 1 87B (PP1) activity had no effect on Orb2A or Orb2B abundance (Figure 5E and Figure S5F). These results suggest like Tob, Orb2 phosphorylation is regulated by PP2A. However, unlike Tob, inhibition of PP2A stabilizes Orb2, particularly Orb2A.

### Tob Promotes LimKinase-Orb2 Association

How does Tob promote Orb2A stabilization and/or enhanced Orb2 oligomerization? Because phosphorylation enhances Orb2 stability, one possibility is that Tob prevents PP2A from accessing Orb2A. However, the association of PP2A catalytic subunit Mts or regulatory subunit Tws with Orb2 was not affected by increased levels of Tob, and the effect of PP2A on Orb2A half-life was not dependent on Tob level (Orb2A, 25.6±14.7 h, *p* = 0.02, and Orb2B, 19.5±8.3, *p* = 0.01) (Figure S6A). However, we found Tob promotes Orb2 phosphorylation by recruiting LimK to Tob-Orb2 complex.

**Figure 6:**
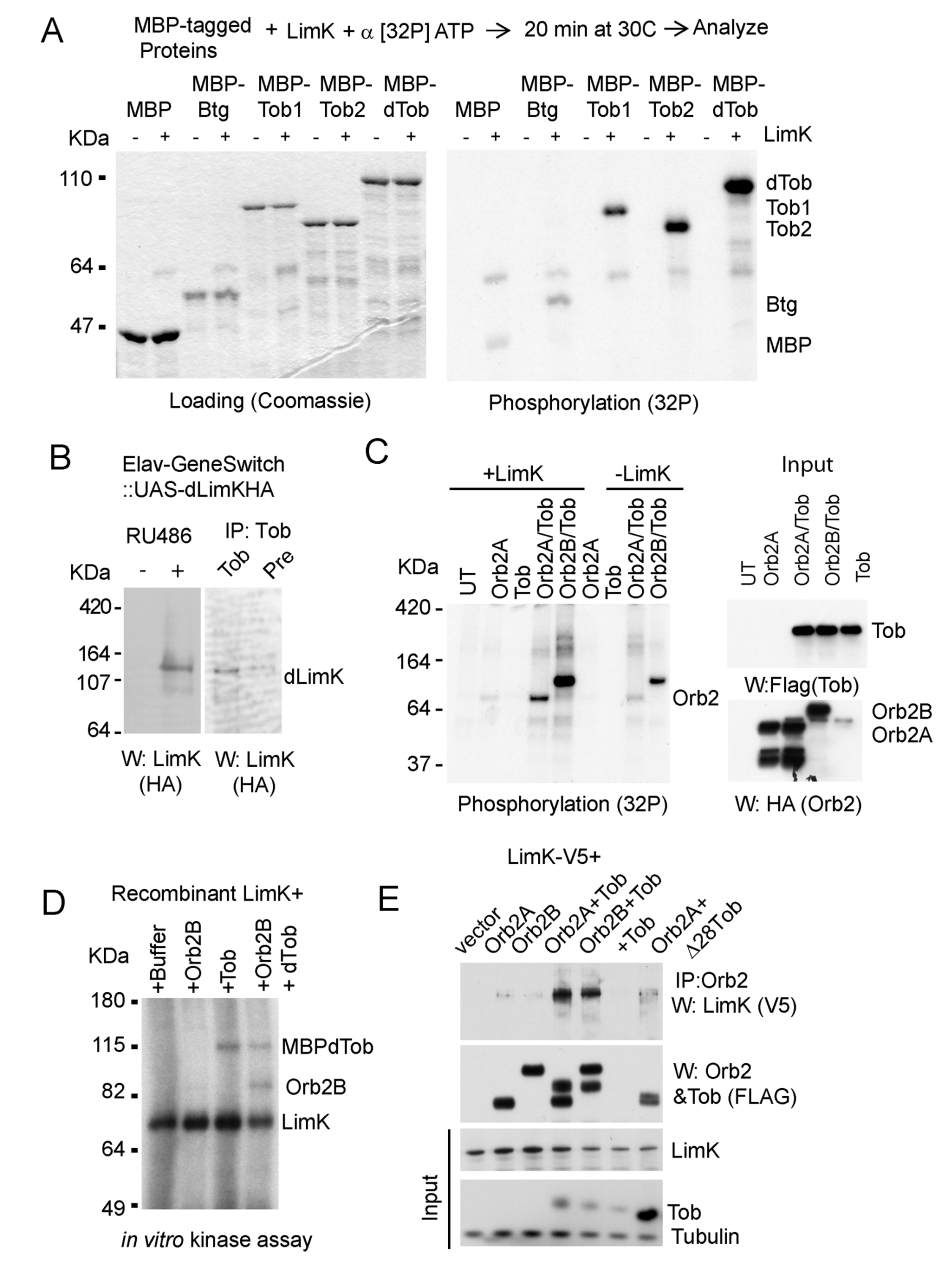
Tob promotes LimK-Orb2 association and Orb2 phosphorylation. (A) Tob is a substrate for Lim kinase. An *in vitro* kinase assay was performed using recombinant maltose binding protein (MBP) and MBP-tagged mammalian Btg, Tob1, Tob2, and *Drosophila* Tob (dTob) as substrates (left panel). *Drosophila* Tob was phosphorylated, as were Tob1 and Tob2, while the more distantly related Btg exhibited background levels of phosphorylation similar to MBP (right panel). The calculated molecular weight of MBP tag is 42.5 KDa and that of MBP-tagged *Drosophila* Tob is ∼101 KDa. (B) Lim kinase interacts with Tob. (Right panel) Tob was immunoprecipitated from adult head extracts expressing HA-tagged LimK under Elav-GeneSwitch inducible driver line and blotted with anti-HA antibodies for LimK. (Left panel) The +RU486 lane contains 5% of the lysate used for immunoprecipitation in the right panel. (C) Lim kinase phosphorylates Tob-associated Orb2A and Orb2B. Orb2 with and without Tob was immunopurified and dephosphorylated prior to use as substrates in the *in vitro* kinase assay. Phosphorylation was assessed in the presence (+LimK) or absence (−LimK) of LimK. Orb2 by itself shows a low level of phosphorylation, suggesting either endogenous LimK or some other kinase is copurified. Phosphorylation significantly increases with the addition of LimK. Western blots on the right show expression levels of the individual components as loading controls. (D) LimKinase phosphorylates recombinant Orb2B in the presence of recombinant MBP-tagged Tob. Due to insolubility, it was not possible to test recombinant Orb2A in this assay. The LimK used in this study is also autophosphorylated. (E) LimKinase (tagged with V5-epitope) forms a complex with Orb2 only in the presence of Tob that interacts with Orb2. In presence of TobΔ28, which interacts weakly with Orb2, only background level of LimK was detected in the Orb2 complex. Also see Figure S6.

In our effort to identify kinases that phosphorylate Tob, we initially focused on MapK, as in mammals and in *C. elegans* Tob is phosphorylated by Map Kinase (MapK) [19],[30],[31] and MapK sites are conserved in *Drosophila* Tob (Figure S6B). However, in an *in vitro* kinase assay, MapK did not phosphorylate recombinant *Drosophila* Tob, although as expected mammalian Tob1 and Tob2 were phosphorylated (Figure S6C). We searched for other kinases and focused on the neuronal kinase LimK for several reasons. First, Tob activity is regulated by BMPs, and in the nervous system LimK is a key mediator of BMP signaling [40]–[44]. Second, neuronal activity regulates the synaptic concentration of LimK [15]. Finally, LimK is required for synapse formation [40],[45],[46], which is reminiscent of the function of ApCPEB [10] and Orb2 (our unpublished observation). In an *in vitro* kinase assay, we found LimK efficiently phosphorylates recombinant *Drosophila* Tob as well as the mammalian Tob1 and Tob2 (Figure 6A) but weakly phosphorylates maltose binding protein or Tob family member Btg. Tob is a LimK substrate because in the adult fly head (Figure 6B) as wells as in S2 cells (Figure S6D) LimK associates with Tob.

Next we sought to determine whether Tob phosphorylation by LimK is influenced by Orb2. We performed *in vitro* LimK assays on immunopurified Tob-Orb2 complex or on Tob alone (Figure 6C). To our surprise, we observed that Orb2 is phosphorylated by exogenously added LimK in the presence of Tob (Figure 6C). The Tob-Orb2 immunoprecipitate from cells contains other proteins in addition to Tob and Orb2, and therefore Orb2 may be phosphorylated by other kinases in the presence of LimK. To test such a possibility, we incubated recombinant-soluble Orb2B protein and LimK in the presence or absence of recombinant MBP-tagged Tob. We observed phosphorylation of Orb2B by LimK in the presence of Tob (Figure 6D). Furthermore, LimK copurified with both Orb2A and B only in the presence of Tob. However, in the presence of TobΔ28, which binds efficiently to LimK (Figure S6D) but not to Orb2, there was a marked reduction in the LimK-Orb2 complex (Figure 6E). Together, these data suggest that Tob is a substrate for LimK and that Orb2 proteins become a substrate of LimK when associated with Tob.

### Lim Kinase Enhances Orb2 Amyloid-Like Oligomerization

**Figure 7:**
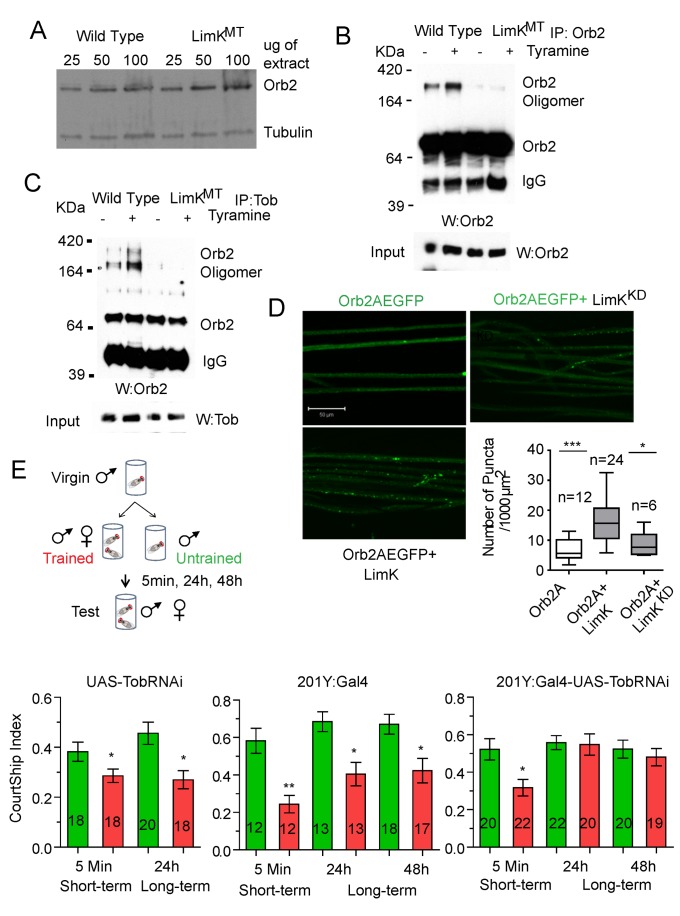
LimK regulates Orb2 oligomerization, and Tob is necessary for long-term memory. (A) Orb2B protein level is not affected in LimK mutant flies. The indicated amounts of total head extracts were blotted with anti-Orb2 antibodies. The same blot was also probed for tubulin as loading control. (B and C) Reduction in LimK activity reduces Orb2 oligomerization in the adult brain. Unlike wild-type flies, very little SDS-resistant Orb2 oligomers were observed in flies with a hypomorphic p-element allele of LimK, *LimK^EY08757^* (LimK^MT^). The extent of Orb2 oligomerization was assessed by immunopurification of Orb2 (B) or Tob-associated Orb2 (C). The level of Orb2 and Tob are shown as input. (D) LimK overexpression increases Orb2AEGFP oligomerization *in vivo*. Orb2AEGFP and either wild-type LimK (LimK) or kinase dead LimK (LimK^KD^) were expressed in the nervous system using Elav-Gal4 driver, and motor neuron axonal projections of third instars larvae were imaged as whole-mount preparations. A point mutation inhibiting LimK activity (D500A, LimK^kd^) is used to control for kinase activity. The addition of active LimK enhances puncta formation as quantified using Axiovision software. Statistical significance was measured with two-tailed *t* test (*) *p*≤0.05, (***) *p*≤0.001. *n*, the number of flies examined for each genotype. Scale bar, 50 µm. Also see Figure S5. (E, Top) A schematic of the male courtship paradigm. (Bottom) Reduction of Tob in mushroom body γ lobe (201Y:Gal4-UAS-TobRNAi) impairs memory at 24 h and 48 h, while 5 min memory remains intact. The heterozygotes control flies for TobRNAi and 201Y:Gal4 show memory at all time points. The numbers indicate number of animals examined in each experimental group. The plots indicate mean courtship index ± SEM: (TobRNAi/+) at 5 min, untrained 0.38±0.04, trained 0.28±0.02; at 24 h, untrained 0.46±0.04; trained 0.27±0.03; (201Y:Gal4/+) at 5 min, untrained 0.58±0.07, trained 0.24±0.05; at 24 h, untrained 0.68±0.05, trained 0.40±0.06; and at 48 h, untrained 0.67±0.05, trained 0.42±0.06; (201Y:Gal4-UAS-TobRNAi) at 5 min, untrained 0.52±0.06, trained 0.31±0.04; at 24 h, untrained 0.56±0.04, trained 0.55±0.06; at 48 h, untrained 0.52±0.05,trained 0.48±0.04. Statistical significance was measured with two-tailed *t* test (*) *p*≤0.05, (**) *p*≤0.01.

Does LimK affect Orb2 oligomerization? To determine whether LimK regulates activity-dependent oligomerization of Orb2 in the adult brain, we examined Orb2 oligomer formation in a LimK hypomorphic mutant *LIMK1^EY08757^* [40]. In the *LIMK1^EY08757^* adult brain, the level of monomeric Orb2B protein level was similar to that of wild-type flies (Figure 7A). We exposed wild-type and LimK mutant flies to 10 mM tyramine and immunopurified either the Orb2 oligomers (Figure 7B) or the Orb2 oligomers associated with Tob (Figure 7C). In the unstimulated brain extract, little or no oligomeric Orb2 was observed in the LimK mutant flies (Figure 7B and C). More importantly, unlike wild-type flies, LimK mutant flies did not undergo a tyramine-dependent increase in Orb2 oligomerization (Figure 7B and C).

To determine whether an increase in LimK activity enhances Orb2 oligomerization, we analyzed Orb2 puncta formation in the larval neuron, where unlike the adult brain, ectopic expression of LimK did not cause any observable developmental problem. We found that Orb2A-EGFP coexpressed with active LimK (ElavGal4::UAS-Orb2A-EGFP; UAS-LimK) has twice the number of puncta (16.10±1.36, *N* = 24) compared with flies coexpressing a kinase dead version of LimK, LimK^KD^ (ELAV::UAS-Orb2A-EGFP; UAS-LimK^KD^) (8.71±1.74, *N* = 6, *p*<0.05) or flies expressing only Orb2A-EGFP (6.79±1.01, *N* = 12, *p*<0.001) (Figure 7D). From these several results, we conclude that Tob serves two functions for Orb2A. First, it binds and stabilizes unphosphorylated Orb2A, and second, it allows Orb2A to be phosphorylated by LimK. Each of these events results in an increase in the effective concentration of Orb2A, which induces Orb2A and/or Orb2A-Orb2B oligomerization.

### Tob Is Required for Long-Term Memory

Because Orb2 oligomerization is important for long-term memory and Tob affects Orb2 oligomerization, we wondered whether Tob activity is important for long-term memory. To this end, we used the male courtship suppression paradigm in which a virgin male fly learns to suppress its courtship behavior upon repeated exposure to an unreceptive female (Figure 7E) [47]. Previously we and others have found male courtship suppression memory is dependent on Orb2 activity [4],[5]. The TobRNAi was expressed under mushroom-body-specific driver 201Y Gal4, which drives expression primarily in the γ-lobe neurons [48]. Expression of Orb2 in γ-lobe in an otherwise *orb2* null background is sufficient to rescue the long-term memory defect [3],[4]. We found that male flies expressing TobRNAi (201Y:Gal4-UAS-TobRNAi) in the γ-lobe showed courtship suppression after training in the short term (5 min), but the courtship suppression was lost when measured at 24 h or 48 h after training (Figure 7E). In contrast, flies harboring just the RNAi (UAS-Tob RNAi) or Gal4 (201Y:Gal4) had no impairment in courtship suppression 5 min or 24 to 48 h after training. These results are consistent with the idea that Tob activity is important for long-term courtship suppression memory.

## Discussion

Our previous work suggested that conversion of neuronal CPEB to an amyloid-like oligomeric state provides a molecular mechanism for the persistence of memory [5],[6]. However, it is not known how Orb2 oligomerization is regulated so that it will occur in a neuron/synapse-specific and activity-dependent manner. Here we report that factors that influence Orb2A stability and thereby abundance regulate Orb2 oligomerization.

We find that Tob, a previously known regulator of SMAD-dependent transcription [23],[24] and CPEB-mediated translation [16], associates with both forms of Orb2, but increases the half-life of only Orb2A. Stimulation with tyramine or activation of mushroom body neurons enhances the association of Tob with Orb2, and overexpression of Tob enhances Orb2 oligomerization. Both Orb2 and Tob are phosphoproteins. Phosphorylation destabilizes Orb2-associated Tob, whereas it stabilizes Orb2A. Tob promotes Orb2 phosphorylation by recruiting LimK, and PP2A controls the phosphorylation status of Orb2A and Orb2B.

PP2A, an autocatalytic phosphatase, is known to act as a bidirectional switch in activity-dependent changes in synaptic activity [14],[49]–[51]. PP2A activity is down-regulated upon induction of long-term potentiation of hippocampal CA1 synapses (LTP) and up-regulated during long-term depression (LTD) [14]. Similarly, Lim Kinase, which is synthesized locally at the synapse [15] in response to synaptic activation, is also critical for long-term changes in synaptic activity and synaptic growth [46].

**Figure 8:**
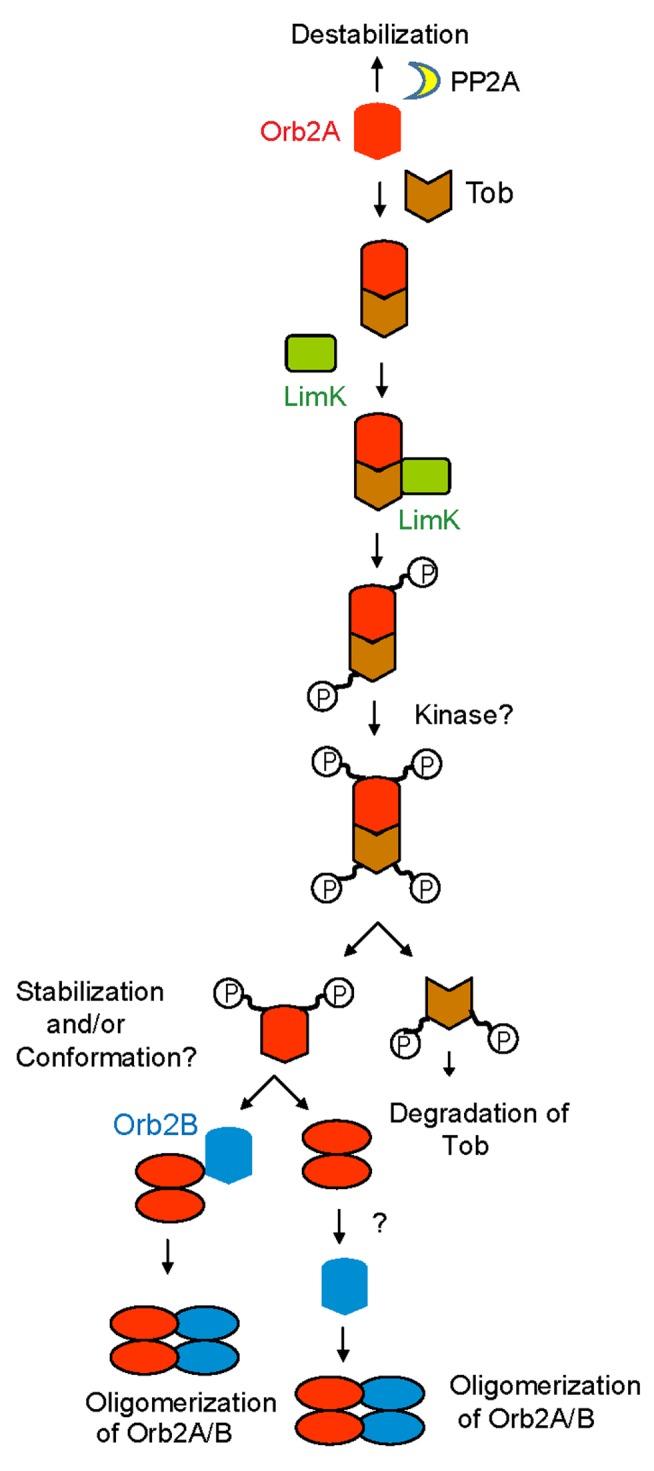
Model of activity-dependent Orb2 oligomerization. The model depicts the sequence of events that leads to oligomerization of Orb2A and destabilization of Tob. PP2A keeps both Orb2 and Tob in an unphosphorylated form, which particularly destabilizes Orb2A. Synaptic activation leads to Orb2A synthesis, and the unphosphorylated Orb2A is bound and stabilized by unphosphorylated Tob. Tob recruits activated LimK to the complex, which phosphorylates Orb2A and Tob. The initial phosphorylation of Tob and Orb2 can trigger additional phosphorylation by other kinases, which leads to the dissociation and destabilization of Tob. On the other hand, the phosphorylation of Orb2A leads to further stabilization and/or changes in conformation to induce oligomerization. The oligomeric Orb2A may directly or indirectly induce oligomerization of Orb2B. The phosphorylation of Orb2A and Orb2B may also regulate the function of both proteins.

Based on these observations we propose a model for activity-dependent and synapse-specific regulation of amyloid-like oligomerization of Orb2 (Figure 8). We postulate that in the basal state synaptic PP2A keeps the available Orb2A in an unphosphorylated and thereby unstable state. Neuronal stimulation results in synthesis of Orb2A by a yet unknown mechanism. The Tob protein that is constitutively present at the synapse binds to and stabilizes the unphosphorylated Orb2A and recruits the activated LimK to the Tob-Orb2 complex, allowing Orb2 phosphorylation. Concomitant decreases in PP2A activity and phosphorylation by other kinases enhances and increases Orb2A half-life. The increase in Orb2A level as well as phosphorylation may induce conformational change in Orb2A, which allows Orb2A to act as a seed. Alternatively, accumulation and oligomerization of Orb2A may create an environment that is conducive to overall Orb2 oligomerization. In the absence of an *in vitro* Orb2A-Orb2B oligomerization assay, we could not distinguish between these two possibilities.

For Tob, initial Orb2 association stabilizes Tob. However, association with Orb2 as well as suppression of PP2A activity leads to additional phosphorylation, which results in dissociation of Tob from the Orb2-Tob complex and destabilization. The destabilization of Orb2-associated Tob provides a temporal restriction to the Orb2 oligomerization process. The coincident inactivation of PP2A and activation of LimK may also provide a mechanism for stimulus specificity and synaptic restriction.

We find that Orb2A and Orb2B are phosphorylated at multiple sites, including serine/threonine and presumably tyrosine residues. These phosphorylation events are likely mediated by multiple kinases because overexpression of LimK did not affect Orb2 phosphorylation to the extent observed with the inhibition or activation of PP2A, raising several interesting questions. In what order do these phosphorylations occur? What function do they serve individually and in combination? What kinases are involved? Moreover, similar to mammalian CPEB family members, in addition to changing stability, phosphorylation may also influence the function of Orb2A and Orb2B.

Does Tob regulate Orb2 function? In mammals Tob has been shown to recruit Caf1 to CPEB3 target mRNA, resulting in deadenylation [16], and CPEB3 is known to act as a translation repressor when ectopically expressed. We find *Drosophila* Tob also interacts with Pop2/Caf1 (Figure S3E) [25] and Orb2A and Orb2B can repress translation of some mRNA [52]. Orb2 has also been identified as a modifier of Fragile-X Mental Retardation Protein (FMRP)–dependent translation, and Fragile-X is believed to act in translation repression [53]. Therefore, the Tob-Orb2 association may contribute to Orb2-dependent translation repression, and the degradation of Orb2-associated Tob may relieve translation repression. Additionally, if the oligomeric Orb2 has an altered affinity for either mRNA or other translation regulators, Tob can affect Orb2 function by inducing oligomerization. However, the relationship between Tob phosphorylation and its function is unclear at this point.

Does involvement of Tob both in transcription and translation serve a specific purpose in the nervous system? Tob inhibits BMP-mediated activation of the Smad-family transcription activators (Smad 1/5/8) by promoting association of inhibitory Smads (Smad 6/7) with the activated receptor [18],[24],[54]–[56]. In *Drosophila* BMP induces synaptic growth via activation of the Smad-family of transcriptional activators, and subsequent stabilization of these newly formed synapses via activation of LimK [57]–[60]. Our studies suggest Tob and LimK also regulate Orb2-dependent translation, raising the possibility Tob may coordinate transcriptional activation in the cell body to translational regulation in the synapse.

## Materials and Methods

### Proteomic Analysis

Please see Text S1 for a detail description of the proteomic analysis.

### *Drosophila* Stocks

The Orb2 lines have been previously described [5],[52]. The following *Drosophila* strains were obtained from Bloomington Stock Center: mts^XE-2258^ (Stock 5684), Pp2A-29B^EP2332^ (Stock 17044), P{EPgy2}LIMK1^EY08757^(Stock 17491), UAS-LimK1HA (Stock 9116), and UAS-LimK1 Kinase dead (Stock 9118). The TobRNAi (Stock 38299) on the second chromosome was obtained from Bloomington TRiP collection. The Gal4 lines were generously provided by Douglas Armstrong (c547-Gal4, c747-Gal4) [61], Troy Zars (MB247, 201Y) [48], and Haig Keshishian (elav-GeneSwitch) [62]. The c547 drives expression primarily in the ellipsoid body, c747, MB247 in all lobes of the mushroom body and 201Y primarily in the γ-lobe of the mushroom body. The elav-GeneSwitch drives expression pan-neuronally in an inducible manner. The UAS-dTrpA1 line was generously provided by Paul Garrity [29]. For expression using the GeneSwitch system, the flies were starved for 16–18 h and then transferred to 2% sucrose containing 200 µM RU486 (mifepristone, SigmaM8046) for 12 h. Various genetic combinations were made by standard genetic crosses.

### Plasmid Constructs

Orb2AHA and Orb2BHA constructs were previously described [5]. The untagged Orb2 and Orb2-interacting protein constructs were made by cloning the full-length PCR products into TopoDonor vector (Invitrogen) and were subsequently transferred to p AWF using the Gateway cloning system (Invitrogen). The *Drosophila* Tob cDNA was amplified by RT-PCR and cloned with Topo-TA (Invitrogen). Flag-tagged Tob was created by the subsequent transfer to the mammalian expression vector, pCMV24 (Invitrogen). Standard molecular techniques were then used to subclone into pMT (Invitrogen) for S2 cell expression and pUAST (DGRC) for use as a *Drosophila* transgene. To create TobΔ28, containing an internal deletion of 28 amino acids (AA235–262), the amino terminal region and C-terminal regions were amplified separately and engineered to contain an internal NotI site. The two fragments (EcoRI/NotI and NotI/SalI) were cloned into pCMV24C. Standard techniques were then used to subclone into pMT and pUAST. For the imaging studies, the tdTomato cDNA was inserted in frame to the C-terminal to create pUAST-TobTdTom and pUAST-TobΔ28TdTom. For antigen production, the cDNA encoding Tob AA 267–564 were amplified by PCR and cloned into pRSETA (Invitrogen) in frame with the 6XHis tag. The mammalian cDNAs for Tob1, Tob2, Ana, and Btg were amplified by RT-PCR from mouse RNA and cloned with Topo-TA, which was subsequently used to produce pCMV24. For production of recombinant proteins in *E. coli*, Tob, Tob1, Tob2, and Btg were reamplified using primers designed to produce an in-frame 6XHis tag at the C-terminus and then subcloned into pMal-c2X. A full-length cDNA encoding LimK, LD15137 was obtained from DGRC and amplified by PCR for Topo TA cloning. The insert was subsequently transferred to pAcV5 for S2 cell expression. LimK^MT^ was engineered to mutate D500K by site-directed mutagenesis (Stratagene). All sequences were confirmed against the NCBI sequence prior to use.

The pCasperOrb2AEGFP construct is comprised of a genomic fragment 1446 nucleotides 3′ of the last Orb2B-specific exon and 1338 bp 5′ of the exonic sequence of the neighboring gene and therefore does not contain coding region of any of the Orb2 isoforms except Orb2A. The ∼8.27 Kb genomic fragment was cloned into the SpeI/XhoI site of pCasper4, and EGFP was introduced at the C-terminal end by creating an in-frame SgrA1 site.

### Cell Culture

Mammalian HEK293 cells were maintained in Dulbecco's modified Eagle's medium supplemented with 10% FBS. Transfections were performed using Lipofectin reagent (Invitrogen). Drosophila S2 cells were maintained in Schneider's medium supplemented with 10% FBS with transfections performed using Effectene reagent (Qiagen). The constructs used are as indicated in the figures. When examining quantitative changes, an empty vector was used to ensure equal quantity of DNA in each transfection. Imagequant software was used to determine densiometric changes, which were subsequently analyzed using Graphpad Prizm software.

### Immunoprecipitation

For immunoprecipitations from cell culture, 3×10^5^ transfected cells were used for each immunoprecipitation. The expression constructs used are as indicated in the figures. Following transfection (36–48 h), the cells were washed in PBS and lysed in 500 µl of 1% Igepal buffer (50 mM Tris-Cl, 7.5, 150 mM NaCl, 1% NP-40 [Igpal], 1 mM DTT, EDTA free protease inhibitor) and clarified by centrifugation at 14,000 rpm for 10 min. For immunoprecipitations from flies, adult heads were collected following flash freezing and vortexing, lysed in 1% Igepal buffer, and clarified by two rounds of centrifugation at 14,000 rpm for 10 min. Protein concentration was determined using a BCA kit (Pierce Biotechnology), and between 1–4 mg of head lysate were used for each immunoprecipitation. The following antibodies were used for immunoprecipitation: anti-HA agarose (Sigma), anti-Flag agarose (Sigma), anti-Tob antibody (raised in guinea pig 2163), and anti-Orb2 (raised in guinea pig-2233 and rabbit-273,402) in conjunction with Protein-A agarose (Repligen). The anti-Tob antibody was raised in guinea pig against the C-terminal end of Tob (Pocono Rabbit Farm), purified using Melon resin (Pierce Biotechnology), and used at 1∶100 dilution. Immunoprecipitations performed using S2 cells were incubated for 2 h at 4°C with continuous rocking, and immunoprecipitations performed using head lysates were incubated for 2 h, and then the ProteinA agarose beads were added with additional 2 h incubation. Following four washes, samples were boiled for 5 min in SDS-PAGE gel loading buffer containing 10% SDS and 2 mM freshly prepared DTT. For immunoprecipitation of Orb2 ∼1 mg of total protein and for Orb2AEGFP ∼3 mg of total protein were used. Western analysis was performed following standard protocols. The following antibodies were used for Western analysis: anti-Flag-HRP (Sigma, 1∶1,000), anti-HA-HRP (Roche, 1∶500), anti-Tob (guinea pig, 1∶1,000), anti-Orb2 (rabbit, 1∶2,000), anti-Orb2 (guinea pig, 1∶1,000), anti-Orb2B (rabbit, 1∶1,000), and anti-EGFP (MBL, 1∶1,000).

### Immunofluorescence

To examine endogenous Tob expression in wild-type CantonS flies and c547-Gal4::UAS-Orb2AEGFP and c547-Gal4::UAS-Orb2BEGFP flies, the proboscis was removed and the flies were decapitated. The heads were fixed for 2 h at 4°C in 4% paraformaldehyde (PFA)/PBS, incubated overnight in 20% sucrose/PBS, followed by 2 h in a 30∶70 mixture of 20% sucrose/PBS and OCT embedding media (Tissue-Tek). The heads were then embedded in 100% OCT, and frontal cryosections were made of 12 µm. The sections were permeabilized in 1% TritonX containing PBS for 5 min followed by 10 min in 0.1% TritonX containing PBS (PBST). The slides were blocked in 10% goat serum containing PBST for 1 h, followed by overnight incubation in 1∶50 dilution of melon-purified (Pierce Biotechnology) anti-Tob (2163) antibody. For the CantonS flies, 1∶50 dilution of nc82 (Developmental Studies Hybridoma Bank) was also added to mark the synaptic regions. Anti–guinea pig Alexa-Fluor 633 (Invitrogen) secondary antibody was used for Tob detection, and anti-mouse Alexa Fluor 488 (Invitrogen) was used for nc-82 detection. Images were acquired at 512×512 pixels with a Zeiss LSM 5.0 confocal microscope as 1 µm Z-stacks. Images shown are projections of 10 slices.

### Aggregate Quantification

To examine changes in aggregate number in the adult Orb2EGFP flies, the whole brain was dissected to remove the exoskeleton and air sacs. The brain was fixed in 4% PFA/PBS for 30 min at room temperature, washed three times with PBST for 10 min, and then the whole brain was mounted. Expression of Orb2EGFP and TobTdTom was driven using the ellipsoid body-specific driver, c547. Images were acquired as above. To quantitate the changes in aggregate number, projections of 20 slices were made for each image centering on the central structure of the ellipsoid body. To examine changes in aggregate number in Lim kinase and Orb2EGFP-expressing animals, third instar larvae were filleted and fixed in 4% PFA/PBS for 10 min at room temperature, washed three times with PBST for 10 min, and mounted. Images of the neurites extending from the ventral ganglia were acquired as described. Projections of 10 slices were made.

Axiovision software (Zeiss, v.4.7.1) was used to quantitate total area, aggregate number, and aggregate size. A commander script was written to identify the region of interest and the puncta within the region. All measurement parameters were kept constant for each image.

### Half-Life Determination

pMT∶FlagTob by itself or in conjunction with pMT∶Orb2AHA or pMT∶Orb2BHA was transfected into S2 cells. Expression Tob and Orb2 were induced by adding 700 µM CuSO4. Following 16 h, the cells were washed and incubated with 50 µg/ml cycloheximide. At the indicated times, samples were collected and later analyzed by Western blot using either anti-Flag or anti-HA antibodies. Densitometric measurements were carried out using ImageQuant and plotted (percent remaining of time zero versus time) using Prism Graphpad 5. The decay curve was fitted using first-order kinetics. To determine the half-life of hyperphosphorylated Tob, a similar analysis was performed with the cells being treated with both cycloheximide and calyculin.

### Kinase Assays

To examine Tob phosphorylation, amylose-bound MBP-tagged proteins were incubated with 5 ng of recombinant LimK (Upstate Biotechnology) and 10 µCi of [γ–^32^P]ATP for 20 min at 30°C with semiconstant shaking. Control reactions were performed identically but in the absence of LimK. Kinase dilution buffer and reaction buffer were prepared according to the manufacturer's specifications. Following phosphorylation, the proteins were washed four times in PBS with 0.1% TritonX and once with PBS prior to loading an 8% SDS/PAGE. Following electrophoresis, the gel was dried and exposed from 4 h to overnight. To examine phosphorylation of recombinant Orb2B, His-tagged Orb2B was expressed in *E. coli* BL21(DE3) using a slow induction protocol, and a low amount of soluble protein was purified in Ni+2 column. Approximately 10 ng of Orb2B, MBP-tagged Tob was used in the kinase reaction.

To examine phosphorylation of the Orb2-Tob complex, 6×10^5^ S2 cells were transfected with pAct∶Orb2AHA or pAct∶Orb2B individually and in combination with pMT∶Tob. The cells were lysed in 1% Igepal buffer (50 mM Tris-Cl, 7.5, 150 mM NaCl, 1% NP-40 [Igpal], 1 mM DTT, EDTA free protease inhibitor) and incubated for 15 min with 50 U/ml CIP. Following centrifugation at 14,000 rpm for 10 min, the supernatant was incubated for 2 h at 4°C with anti-HA agarose. The immunoprecipitates were washed twice with 1% Igepal buffer and once with a modified RIPA buffer (50 mM Tris, 300 mM NaCl, 0.1% SDS, 1% Igepal). The sample was then split into thirds, with one-third examined by Western blot to ensure equality in protein levels and the other two-thirds used for the *in vitro* kinase assay described above. For the Tob alone samples, 12×10^5^ S2 cells were transfected with pAcOrb2AHA and pMTTob, and the complex was purified as above and dissociated in 1% Igepal buffer containing 1 M NaCl for 15 min at room temperature. The eluate was then normalized to 150 M NaCl and Tob purified by precipitation with anti-Flag agarose (Sigma). Complete dissociation was ensured by Western analysis.

### Detection of Phosphoprotein

The protein abundance studies were carried out in 4%–12% Bis-Tris SDS-PAGE (Invitrogen) and run in MES-SDS (50 mM MES, 50 mM Tris Base, 0.1% SDS, 1 mM EDTA, pH 7.3) buffer. In these buffer conditions and in the gradient gel, the phosphorylated bands migrate close to each other, which simplifies the quantification of band intensity. Also, in protein abundance studies, the total cell lysates were prepared, unless mentioned, in the absence of phosphatase inhibitors, again to ensure quantification of the total protein accurately. The 4%–12% gels were also used for the detection oligomeric Orb2 and phospho-tag™ blotting of Orb2- or Tob immunoprecipitate from the adult fly head.

To measure phosphorylation status via mobility shift, we found that an 8% SDS-PAGE run in conventional Tris-Glycine buffer (25 mM Tris, 192 mM glycine, 0.1% SDS, pH 8.6) is more effective, and in 8% gel the different phosphorylated forms of Orb2 and Tob proteins were better separated. For detection of the phosphoproteins via phospho-tag™, both 8% and 4%–12% SDS-PAGE were used.

### Male Courtship Suppression Assay

Flies were maintained using standard fly husbandry methods. For behavioral analysis, flies were maintained on standard cornmeal food at 25°C and 60% relative humidity on a 12 h/12 h light-dark cycle. Virgin males and females were collected at eclosion under CO_2_ anesthesia. Males were isolated and placed in individual food vials. All flies were aged for 5 d before behavioral training and testing. To increase the efficiency of RNAi, flies were shifted to 30°C for 3 d before training. The control flies were treated similarly. Canton S females (4 d old) were mated the night before they were used in training. Males were assayed for courtship conditioning using a modified version of the spaced training described by McBride et al. (1999) [63]. For spaced training, individual males were placed in individual small food tubes (16×100 mm culture tubes, VWR) with a mated female for 2 h. The female was removed, and the male was left alone for 30 min. A different mated female was placed in the tube with the male for another 2 h. The female was removed and the male again rested for another 30 min. A third mated female was introduced in the tube for 2 h and removed at the end of the trial. Control males were treated exactly the same way, except no mated females were introduced into the tube. Memory was tested 5 min, 24 h, and 48 h after training. All tests were performed in a 1 cm courtship chamber. Fresh mated females were used for all time points. All memory tests were recorded (for 10 min) and analyzed using a customized software. The courtship index of each male was obtained by manual and/or automatic analysis of the movies by an experimenter blind to the genotype and experimental conditions.

## Supporting Information

Figure S1 **List of Orb2 interacting proteins in the adult fly head (related to** **Figure 1** **).** (A) The list of 61 proteins from the adult fly brain that were significantly enriched in the Orb2 immunoprecipitate over control. The distributions of proteins in various groups are color coded for ease of visualization. (B) Pair wise interaction study of Orb2 and candidate proteins. Representative examples of Orb2A (left panel) and Orb2B (right panel) interaction with candidate proteins. The candidate proteins were expressed in S2 cells as FLAG-tagged protein with untagged Orb2 and immunoprecipitated with anti-FLAG antibodies. Trip1 is a component of translation initiation factor 3 protein complex, and CG32016 is predicted to be an eIF4E regulator. Orb2 interacts with both eIF3 and eIF4E, and therefore the modest binding of Trip1 and CG32016 could be due to their presence in eIF3 or eIF4E protein complexes, respectively. The CG17838 is significantly enriched in Orb2 immunoprecipitate, but does not efficiently form complex with Orb2A. The proteins in the left panel belong to the group of proteins that are enriched in the Orb2 IP, but do not show statistical significance (please see Table S1).
(TIF)

Figure S2 **Conservation of Tob-CPEB interaction (related to** **Figure 2** **).** (A) Reduction in endogenous Tob destabilizes Orb2A. (Left panel) S2 cells transfected with untagged Orb2A or Orb2B were treated with Tob RNAi. After 3 d, steady state level was measured. The asterisk indicates the RNA binding protein hrp36, which is used as a loading control. (Right panel) In S2 cells, double-stranded RNA against Tob reduces endogenous Tob RNA level 3 d posttransfection. GAPDH serves as loading control for RT-PCR. (B) Tob directly interacts with Orb2. *In vitro* pull-down assay was performed using recombinant MBP protein (−) or MBP-tagged Tob (+) and ^35^S-Methionine labeled Orb2A and Orb2B. The autoradiogram is shown in left and the coomassie stained protein gel in the right. (C) Mammalian Tob2 interacts with mouse CPEB3 and *Aplysia* CPEB (ApCPEB). Immunoprecipitations were performed from HEK293T cell extracts transfected with HA-tagged mouse CPEB3 or *Aplysia* CPEB (ApCPEB) together with either flag-tagged mouse Tob1 (mTob1) or mouse Tob2 (mTob2).
(TIF)

Figure S3 **Mapping of Orb2 interacting domain in Tob (related to** **Figure 2** **).** (A) Tob family members are defined by an antiproliferative domain (APRO) consisting of two highly conserved sequences represented by box A and box B. A third conserved sequence with unknown function (box C) is deleted in TobΔ28. (B and C) A conserved 28 amino acid domain is critical for Orb2 binding. TobΔ28 exhibits reduced binding for Orb2 in S2 cells (B) as well as in the adult fly heads (C). (D) The Orb2 interacting domain is not required for Tob interactions with the deadenylase Pop2 or (E) with the *Drosophila* homologue of the transcription factor Smad1, Mad. (F) Tob associates with Orb2A and Orb2B oligomers in the adult fly brain. Tob was immunoprecipitated from adult head extracts prepared from wild-type flies or flies expressing Orb2AEGFP or Orb2B∶EGFP under the neuron-specific Drl-Gal4 driver. Both the monomeric and oligomeric forms of the EGFP-tagged Orb2 proteins are observed in the Tob immunoprecipitates. (G) Overexpression of Tob increases Orb2AEGFP but not Orb2BEGFP puncta. EGFP-tagged Orb2 was expressed in the ellipsoid body using c547-Gal4 with or without TdTomato-tagged Tob. Each row represents a fly genotype: c547-Gal4: UAS-Orb2AEGFP (Orb2A only), c547-Gal4: UAS-Orb2AEGFP/UAS-TobTdtomato (Orb2A Tob),c547-Gal4: UAS-Orb2BEGFP (Orb2B only), c547-Gal4: UAS-Orb2BEGFP/UAS-TobTdtomato (Orb2B Tob), and c547-Gal4: UAS-Orb2AEGFP/UAS-TobΔ28Tdtomato (Orb2A TobΔ28). Higher magnification images of the boxed region are shown in the right. Scale bar, 20 µm.
(TIF)

Figure S4 **Subcellular distribution of Tob (related to** **Figure 3** **).** (A) The specificity of anti-*Drosophila* Tob antibody. Total adult head extracts from Elav-Gal4 or Elav-Gal4:UAS-TobRNAi were Western blotted using anti-Tob antibodies. The position of Tob is indicated. The blot was overexposed to ensure detection of all immunoreactive bands. Tubulin serves as a loading control. (B) Tyramine enhances Tob-Orb2 association. Tob was immunoprecipitated from unstimulated (control), tyramine, or serotonin (5-HT) stimulated head extracts and blotted with the anti-Orb2 antibody. The preimmune (pre) serum from the same animal serves as control for Tob antibody specificity. Western analysis of lysates indicates the expression levels of Orb2, Tob, and tubulin. (C) Tob is present in the cell body and low level in the synaptic neuropil region in the adult *Drosophila* brain. We stained 12 µm thick frontal sections with preimmune or anti-Tob serum. Nc82 stains the synaptic region. The representative image of the antennal lobe region is shown. Scale bar, 20 µm. (D) Tob shows relative enrichment in the synaptic membrane fraction. (Left panel) A schematic depiction of the fractionation procedure used to obtain synaptic membrane and soluble fractions. (Right panel) The Western blot analysis of 50 µg of synaptic membrane or synaptic soluble fraction proteins with antibodies against indicated proteins. Δ80QOrb2 flies lack the n-terminal prion-like domain and have a reduced level of Orb2 protein. (E) Mammalian Tob is present in synaptic membrane fraction. (Left panel) The schematic representation of the synaptosome preparation from adult mouse brain. The fractions blotted for mouse Tob are indicated in red. (Right panel) The antibody recognizes both Tob1 and Tob2. The transcription factor CREB serves as a marker for the nuclear fraction. The metabotropic glutamate receptor Glur1 is a marker for synaptic membrane and SNAP25 and synaptophysin serve as marker for synaptic vesicle fraction.
(TIF)

Figure S5 **Protein phosphatase 2A, but not protein phosphatase 1, regulates Tob and Orb2 (related to** **Figure 5** **).** (A) Tob is stable when coexpressed with Orb2A but becomes hyperphosphorylated and destabilized when the cells are treated with the PP1/PP2A inhibitor, calyculin (CY), or the PP2A specific inhibitor Okadaic acid (OA). Unlike PP2A inhibitors, the PP1 inhibitor tautomycin (TM) had a modest effect on Tob phosphorylation or stability. The plot on the right depicts percent of Tob remaining following treatment with various phosphatase inhibitors. (B) Tob interacts with Orb1 and Orb2 proteins. Flag-tagged Tob was immunoprecipitated from cells transfected with HA-tagged Orb1 and Orb2. (C, top panel) In S2 cells Tob is phosphorylated when coexpressed with Orb2A or Orb2B but not the closely related Orb1. Changes in Tob phosphorylation status was assessed as an increase in molecular weight as determined by Western blot analysis of transfected S2 cells. (Bottom panel) Treatment with λ-phosphatase resulted in reduced size of Tob when coexpressed with Orb2A and Orb2B, but not Orb1. The proteins were analyzed in 4%–12% gel. (D) PP2A inhibitors CY and okadaic acid but not PP1 inhibitor tautomycin enhance Orb2A half-life. (E) The catalytic subunit of PP2A, Mts, associates with Orb2A and Orb2B. The Orb2 proteins were coexpressed with HA-tagged Mts, and the Mts-Orb2 complex was immunopurified with anti-HA antibodies. Because PP2A destabilizes Orb2A and Orb2B, both Orb2 proteins are expressed at a low level in the presence of Mts. (F, top panel) Overexpression of PP2A (+Mts), but not PP1 (+PP187B), destabilizes Orb2A (left panel) and Orb2B (right panel). (Bottom panel) Overexpression of PP2A (+Mts+CY) but not PP1 (+PP187B+CY) reverses the effect of calyculin A. The RNA binding protein Hrp36 serves as loading control.
(TIF)

Figure S6 **Phosphorylation of Tob (related to** **Figure 6** **).** (A) Tob does not affect PP2A-mediated Orb2A stability. The plot depicts percent of Orb2A remaining following treatment with phosphatase inhibitor calyculinA in the presence or absence of Tob. (B) MapK phosphorylation sites (highlighted serine residues) identified in mammalian Tob1 and Tob2 are conserved in *Drosophila* Tob. ClustalV was used to align the three proteins; only the residues encompassing the MapK site are shown. (C) *Drosophila* Tob is not phosphorylated by MapK. An *in vitro* MapK kinase assay using recombinant MBP-tagged mammalian APRO proteins, Btg, Tob1, Tob2, and *Drosophila* Tob (left panel). Phosphorylation is only observed with mammalian Tob1 and Tob2 (right panel). (D) TobΔ28 associates with LimK. V5-tagged LimK was coexpressed with either full-length Tob or TobΔ28. The Tob was immunoprecipitated with Tob preimmune (pre) or immune serum and blotted with anti-V5 antibodies for LimK. We have noticed that TobΔ28 expression level is usually higher than that of full-length Tob.
(TIF)

Table S1 **Proteins detected by MudPIT analysis of Orb2 using Orb2-specific immunopurification and HA immunopurification (related to** **Figure 1** **).**
(XLSX)

Table S2 **Orb2 and Tob stability (related to** **Figure 2** **and** **Figure 5** **).** Orb2 and Tob half-lives were determined by plotting the percent of protein remaining following the addition of 50 µg/ml CHX at select time points and assuming first order kinetics. Each time course was performed a minimum of four times. All data are presented as mean ± SEM, and *p*<0.05 indicates statistical significance, and NS stands for no statistical significance.
(DOCX)

Text S1 **Extended materials and methods.**
(DOCX)

## Abbreviations

BMP: Bone morphogenetic protein
CHX: Cycloheximide
CPEB: Cytoplasmic Polyadenylation Element Binding
CY: CalyculinA
LimK: Lim Kinase
MapK: Mitogen activated protein kinase
PP1: Protein phosphatase 1
PP2A: Protein Phosphatase 2A
Tob: Transducer of Erb-B2
