# Supplementary figures and images for: Contribution of Orb2A Stability in Regulated Amyloid-Like Oligomerization of Drosophila Orb2
Source: PLoS Biol. 2014 Feb 11;12(2):e1001786. doi: 10.1371/journal.pbio.1001786 (PMC3921104; doi:10.1371/journal.pbio.1001786)

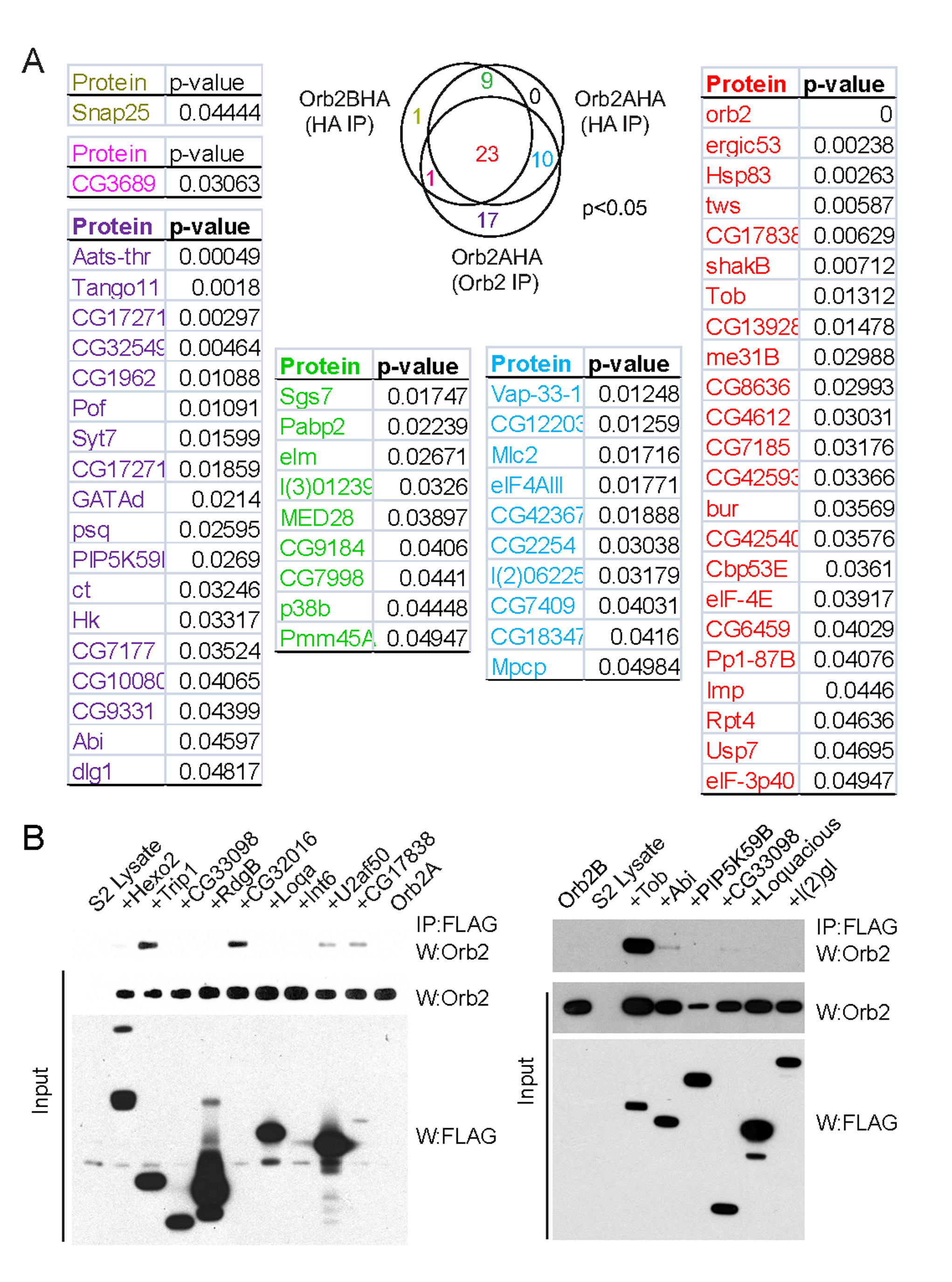

Supplement: Figure S1 — List of Orb2 interacting proteins in the adult fly head (related to Figure 1 ). (A) The list of 61 proteins from the adult fly brain that were significantly enriched in the Orb2 immunoprecipitate over control. The distributions of proteins in various groups are color coded for ease of visualization. (B) Pair wise interaction study of Orb2 and candidate proteins. Representative examples of Orb2A (left panel) and Orb2B (right panel) interaction with candidate proteins. The candidate proteins were expressed in S2 cells as FLAG-tagged protein with untagged Orb2 and immunoprecipitated with anti-FLAG antibodies. Trip1 is a component of translation initiation factor 3 protein complex, and CG32016 is predicted to be an eIF4E regulator. Orb2 interacts with both eIF3 and eIF4E, and therefore the modest binding of Trip1 and CG32016 could be due to their presence in eIF3 or eIF4E protein complexes, respectively. The CG17838 is significantly enriched in Orb2 immunoprecipitate, but does not efficiently form complex with Orb2A. The proteins in the left panel belong to the group of proteins that are enriched in the Orb2 IP, but do not show statistical significance (please see Table S1). (TIF) [file pbio.1001786.s001.tif]

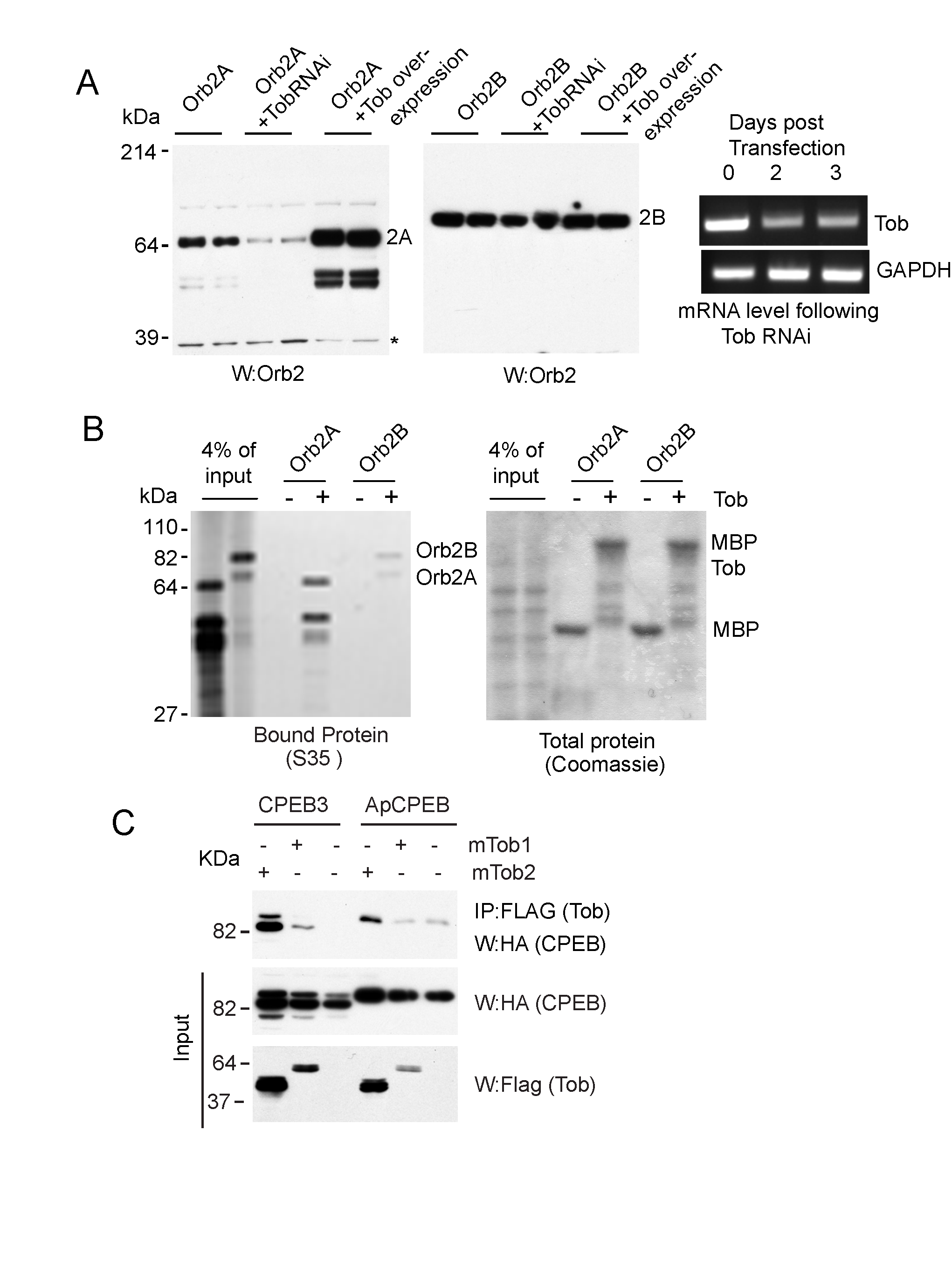

Supplement: Figure S2 — Conservation of Tob-CPEB interaction (related to Figure 2 ). (A) Reduction in endogenous Tob destabilizes Orb2A. (Left panel) S2 cells transfected with untagged Orb2A or Orb2B were treated with Tob RNAi. After 3 d, steady state level was measured. The asterisk indicates the RNA binding protein hrp36, which is used as a loading control. (Right panel) In S2 cells, double-stranded RNA against Tob reduces endogenous Tob RNA level 3 d posttransfection. GAPDH serves as loading control for RT-PCR. (B) Tob directly interacts with Orb2. In vitro pull-down assay was performed using recombinant MBP protein (−) or MBP-tagged Tob (+) and 35S-Methionine labeled Orb2A and Orb2B. The autoradiogram is shown in left and the coomassie stained protein gel in the right. (C) Mammalian Tob2 interacts with mouse CPEB3 and Aplysia CPEB (ApCPEB). Immunoprecipitations were performed from HEK293T cell extracts transfected with HA-tagged mouse CPEB3 or Aplysia CPEB (ApCPEB) together with either flag-tagged mouse Tob1 (mTob1) or mouse Tob2 (mTob2). (TIF) [file pbio.1001786.s002.tif]

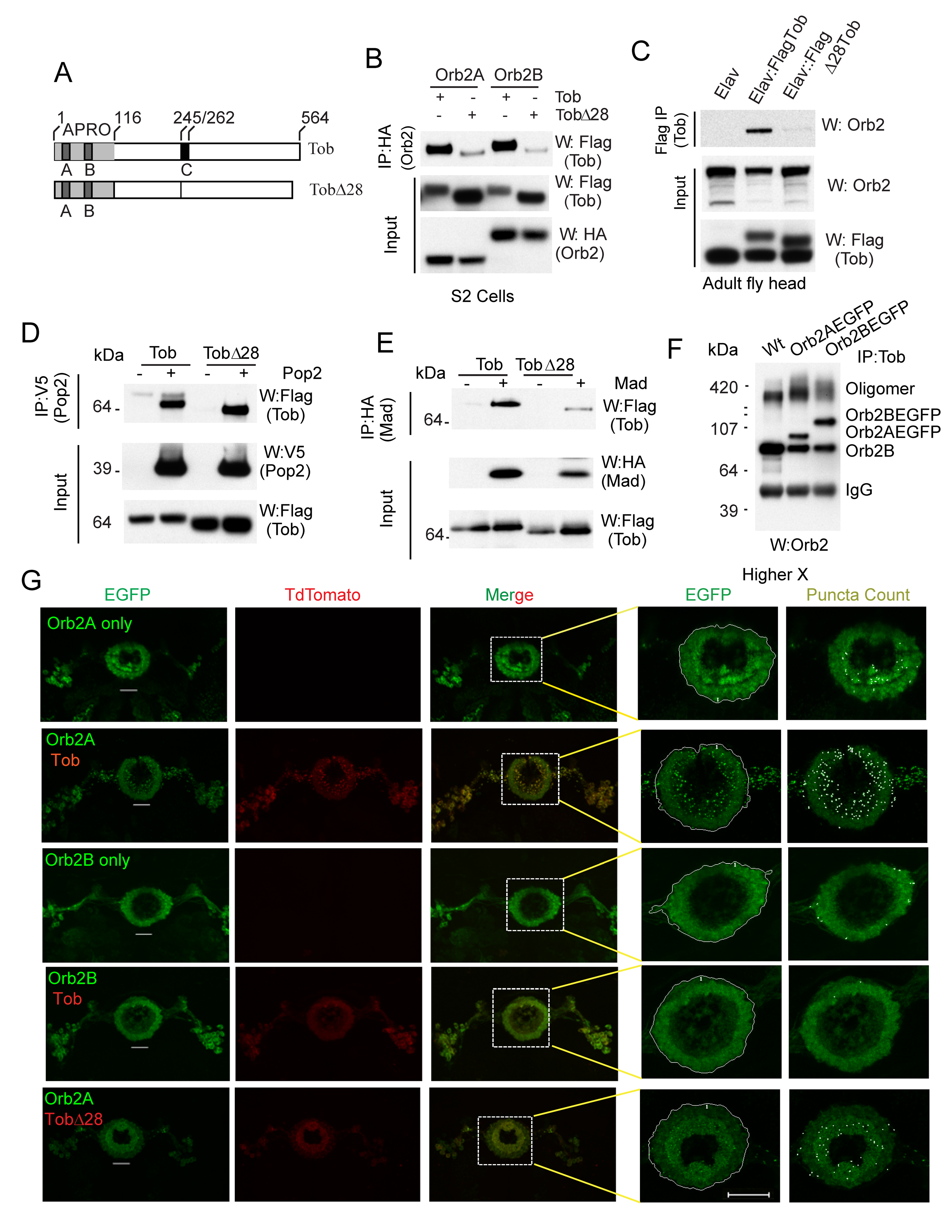

Supplement: Figure S3 — Mapping of Orb2 interacting domain in Tob (related to Figure 2 ). (A) Tob family members are defined by an antiproliferative domain (APRO) consisting of two highly conserved sequences represented by box A and box B. A third conserved sequence with unknown function (box C) is deleted in TobΔ28. (B and C) A conserved 28 amino acid domain is critical for Orb2 binding. TobΔ28 exhibits reduced binding for Orb2 in S2 cells (B) as well as in the adult fly heads (C). (D) The Orb2 interacting domain is not required for Tob interactions with the deadenylase Pop2 or (E) with the Drosophila homologue of the transcription factor Smad1, Mad. (F) Tob associates with Orb2A and Orb2B oligomers in the adult fly brain. Tob was immunoprecipitated from adult head extracts prepared from wild-type flies or flies expressing Orb2AEGFP or Orb2B∶EGFP under the neuron-specific Drl-Gal4 driver. Both the monomeric and oligomeric forms of the EGFP-tagged Orb2 proteins are observed in the Tob immunoprecipitates. (G) Overexpression of Tob increases Orb2AEGFP but not Orb2BEGFP puncta. EGFP-tagged Orb2 was expressed in the ellipsoid body using c547-Gal4 with or without TdTomato-tagged Tob. Each row represents a fly genotype: c547-Gal4: UAS-Orb2AEGFP (Orb2A only), c547-Gal4: UAS-Orb2AEGFP/UAS-TobTdtomato (Orb2A Tob),c547-Gal4: UAS-Orb2BEGFP (Orb2B only), c547-Gal4: UAS-Orb2BEGFP/UAS-TobTdtomato (Orb2B Tob), and c547-Gal4: UAS-Orb2AEGFP/UAS-TobΔ28Tdtomato (Orb2A TobΔ28). Higher magnification images of the boxed region are shown in the right. Scale bar, 20 µm. (TIF) [file pbio.1001786.s003.tif]

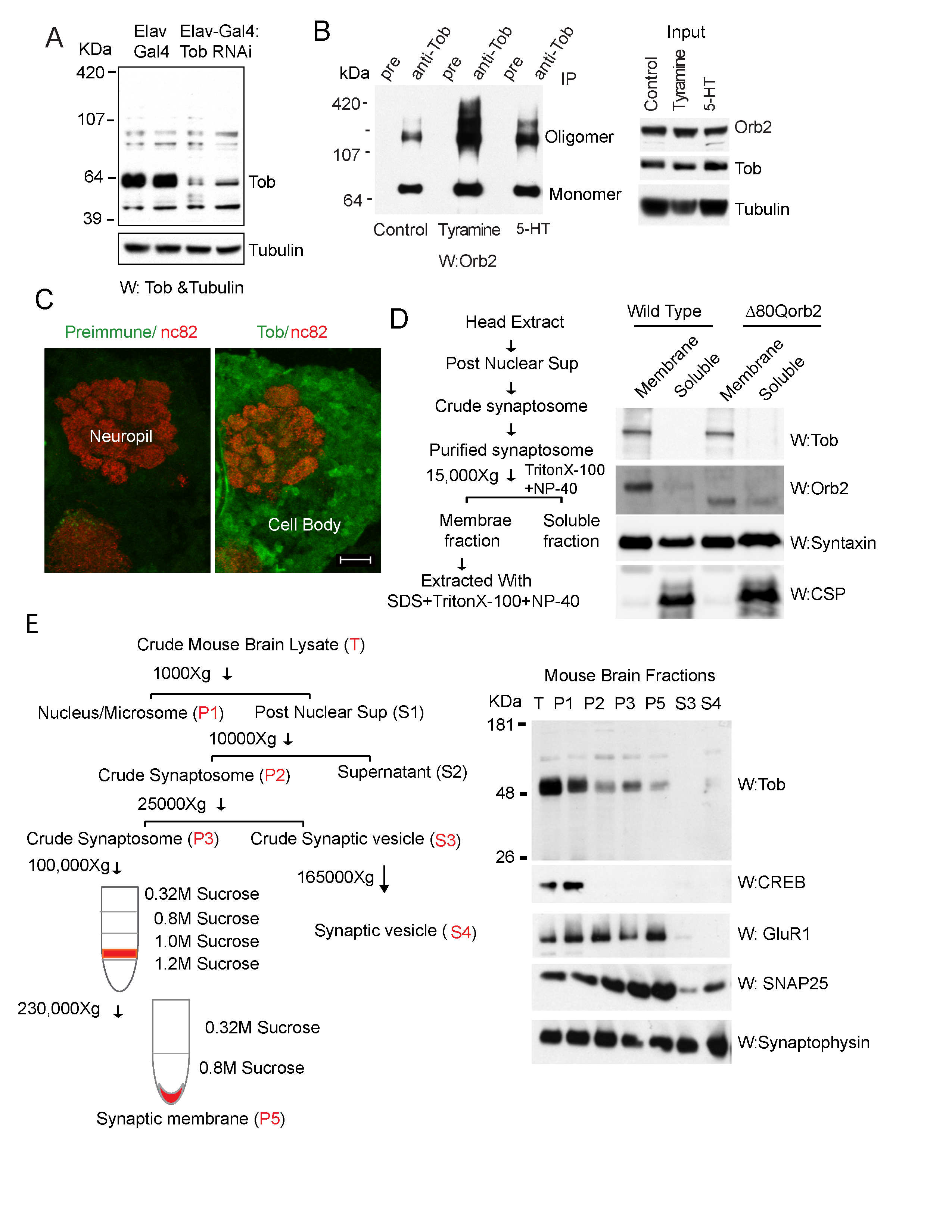

Supplement: Figure S4 — Subcellular distribution of Tob (related to Figure 3 ). (A) The specificity of anti-Drosophila Tob antibody. Total adult head extracts from Elav-Gal4 or Elav-Gal4:UAS-TobRNAi were Western blotted using anti-Tob antibodies. The position of Tob is indicated. The blot was overexposed to ensure detection of all immunoreactive bands. Tubulin serves as a loading control. (B) Tyramine enhances Tob-Orb2 association. Tob was immunoprecipitated from unstimulated (control), tyramine, or serotonin (5-HT) stimulated head extracts and blotted with the anti-Orb2 antibody. The preimmune (pre) serum from the same animal serves as control for Tob antibody specificity. Western analysis of lysates indicates the expression levels of Orb2, Tob, and tubulin. (C) Tob is present in the cell body and low level in the synaptic neuropil region in the adult Drosophila brain. We stained 12 µm thick frontal sections with preimmune or anti-Tob serum. Nc82 stains the synaptic region. The representative image of the antennal lobe region is shown. Scale bar, 20 µm. (D) Tob shows relative enrichment in the synaptic membrane fraction. (Left panel) A schematic depiction of the fractionation procedure used to obtain synaptic membrane and soluble fractions. (Right panel) The Western blot analysis of 50 µg of synaptic membrane or synaptic soluble fraction proteins with antibodies against indicated proteins. Δ80QOrb2 flies lack the n-terminal prion-like domain and have a reduced level of Orb2 protein. (E) Mammalian Tob is present in synaptic membrane fraction. (Left panel) The schematic representation of the synaptosome preparation from adult mouse brain. The fractions blotted for mouse Tob are indicated in red. (Right panel) The antibody recognizes both Tob1 and Tob2. The transcription factor CREB serves as a marker for the nuclear fraction. The metabotropic glutamate receptor Glur1 is a marker for synaptic membrane and SNAP25 and synaptophysin serve as marker for synaptic vesicle fraction. (TIF) [file pbio.1001786.s004.tif]

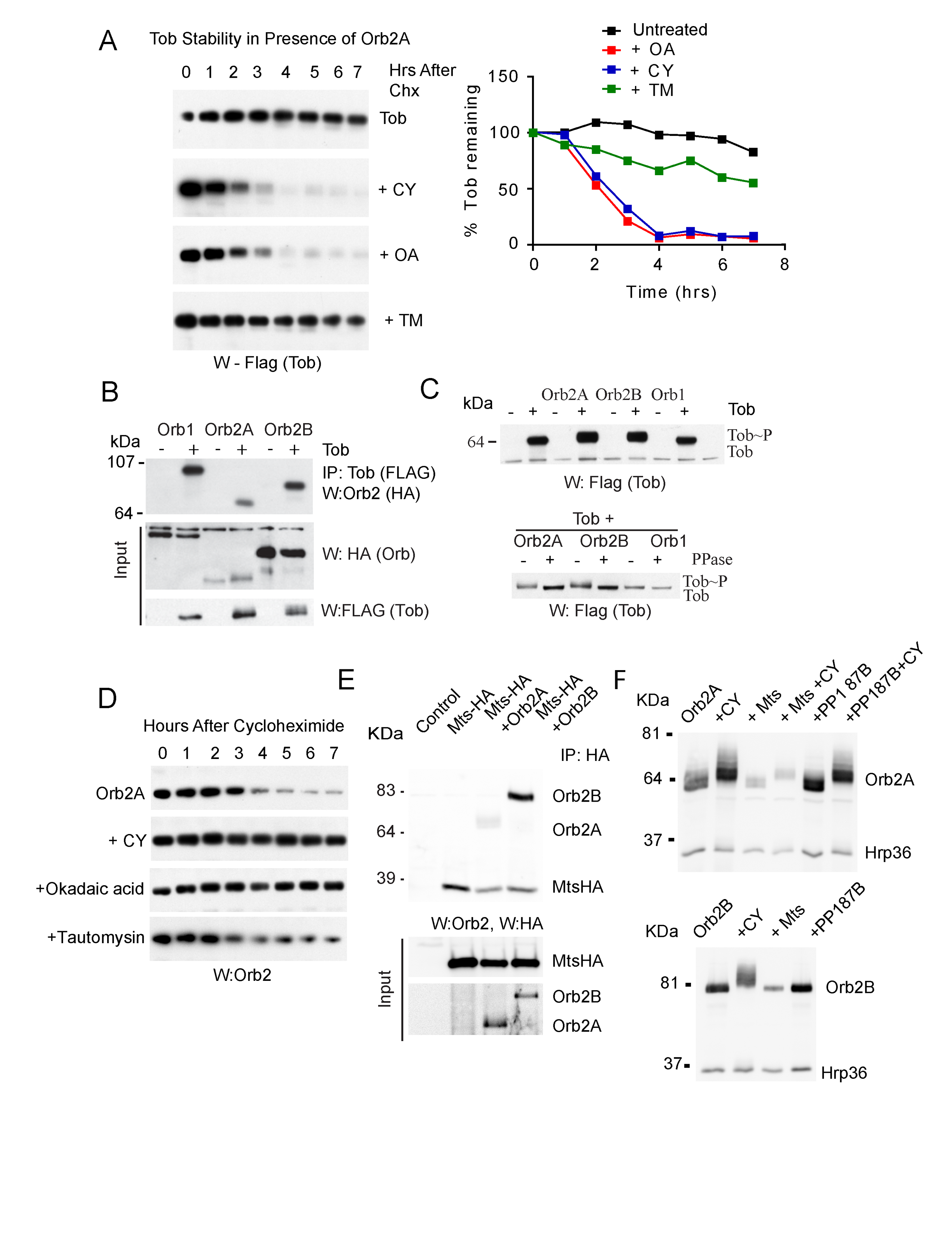

Supplement: Figure S5 — Protein phosphatase 2A, but not protein phosphatase 1, regulates Tob and Orb2 (related to Figure 5 ). (A) Tob is stable when coexpressed with Orb2A but becomes hyperphosphorylated and destabilized when the cells are treated with the PP1/PP2A inhibitor, calyculin (CY), or the PP2A specific inhibitor Okadaic acid (OA). Unlike PP2A inhibitors, the PP1 inhibitor tautomycin (TM) had a modest effect on Tob phosphorylation or stability. The plot on the right depicts percent of Tob remaining following treatment with various phosphatase inhibitors. (B) Tob interacts with Orb1 and Orb2 proteins. Flag-tagged Tob was immunoprecipitated from cells transfected with HA-tagged Orb1 and Orb2. (C, top panel) In S2 cells Tob is phosphorylated when coexpressed with Orb2A or Orb2B but not the closely related Orb1. Changes in Tob phosphorylation status was assessed as an increase in molecular weight as determined by Western blot analysis of transfected S2 cells. (Bottom panel) Treatment with λ-phosphatase resulted in reduced size of Tob when coexpressed with Orb2A and Orb2B, but not Orb1. The proteins were analyzed in 4%–12% gel. (D) PP2A inhibitors CY and okadaic acid but not PP1 inhibitor tautomycin enhance Orb2A half-life. (E) The catalytic subunit of PP2A, Mts, associates with Orb2A and Orb2B. The Orb2 proteins were coexpressed with HA-tagged Mts, and the Mts-Orb2 complex was immunopurified with anti-HA antibodies. Because PP2A destabilizes Orb2A and Orb2B, both Orb2 proteins are expressed at a low level in the presence of Mts. (F, top panel) Overexpression of PP2A (+Mts), but not PP1 (+PP187B), destabilizes Orb2A (left panel) and Orb2B (right panel). (Bottom panel) Overexpression of PP2A (+Mts+CY) but not PP1 (+PP187B+CY) reverses the effect of calyculin A. The RNA binding protein Hrp36 serves as loading control. (TIF) [file pbio.1001786.s005.tif]

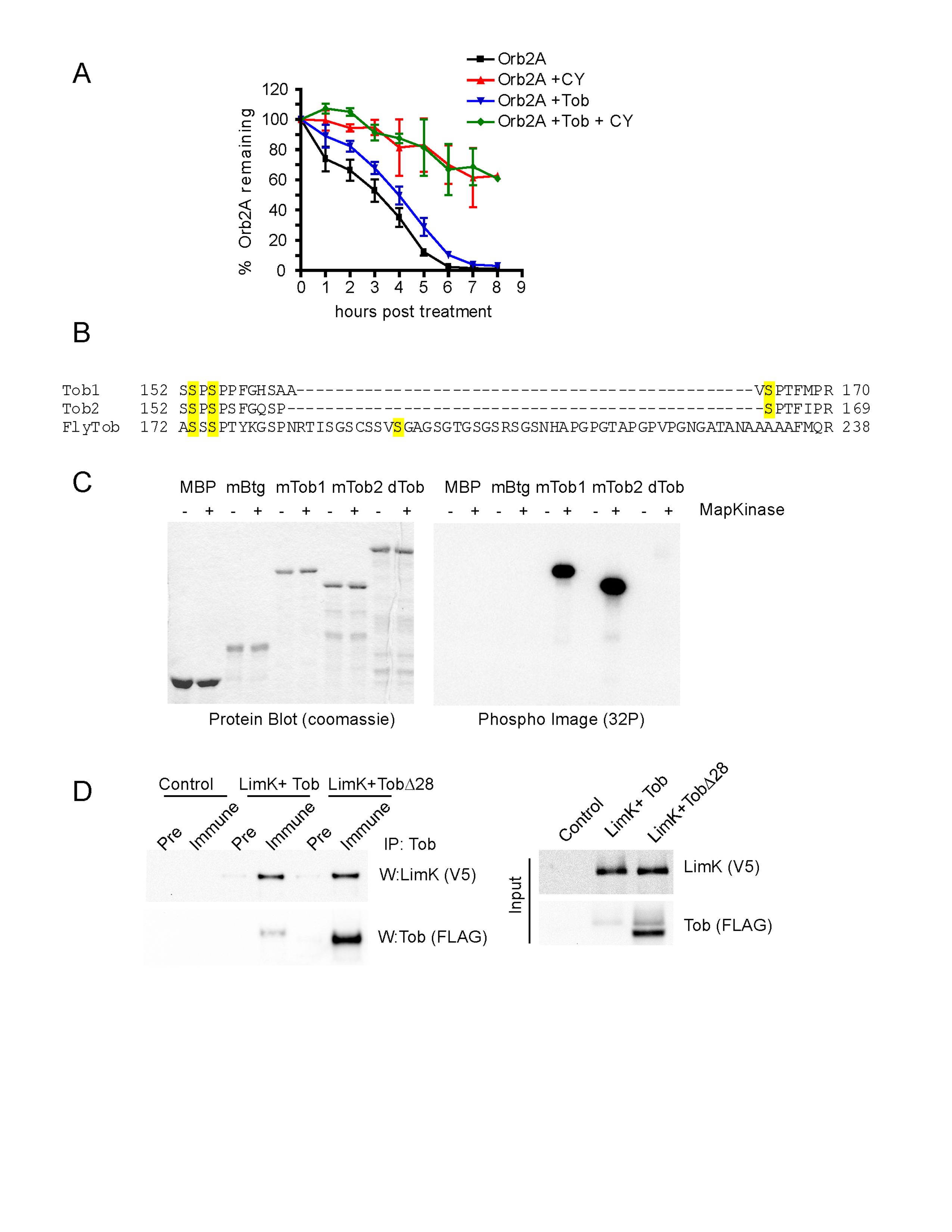

Supplement: Figure S6 — Phosphorylation of Tob (related to Figure 6 ). (A) Tob does not affect PP2A-mediated Orb2A stability. The plot depicts percent of Orb2A remaining following treatment with phosphatase inhibitor calyculinA in the presence or absence of Tob. (B) MapK phosphorylation sites (highlighted serine residues) identified in mammalian Tob1 and Tob2 are conserved in Drosophila Tob. ClustalV was used to align the three proteins; only the residues encompassing the MapK site are shown. (C) Drosophila Tob is not phosphorylated by MapK. An in vitro MapK kinase assay using recombinant MBP-tagged mammalian APRO proteins, Btg, Tob1, Tob2, and Drosophila Tob (left panel). Phosphorylation is only observed with mammalian Tob1 and Tob2 (right panel). (D) TobΔ28 associates with LimK. V5-tagged LimK was coexpressed with either full-length Tob or TobΔ28. The Tob was immunoprecipitated with Tob preimmune (pre) or immune serum and blotted with anti-V5 antibodies for LimK. We have noticed that TobΔ28 expression level is usually higher than that of full-length Tob. (TIF) [file pbio.1001786.s006.tif]
