# Supplementary material for: Contribution of Orb2A Stability in Regulated Amyloid-Like Oligomerization of Drosophila Orb2
Source: PLoS Biol. 2014 Feb 11;12(2):e1001786. doi: 10.1371/journal.pbio.1001786 (PMC3921104; doi:10.1371/journal.pbio.1001786)
Supplement: Table S2 — Orb2 and Tob stability (related to Figure 2 and Figure 5 ). Orb2 and Tob half-lives were determined by plotting the percent of protein remaining following the addition of 50 µg/ml CHX at select time points and assuming first order kinetics. Each time course was performed a minimum of four times. All data are presented as mean ± SEM, and p<0.05 indicates statistical significance, and NS stands for no statistical significance. (DOCX) [file pbio.1001786.s008.docx]

| **Table S2. Orb2 and Tob Stability** | | |
| --- | --- | --- |
| **Half-life (Hours)** | **Half-life (Hours)** | p Value |
| **Orb2A stability** | | |
| Orb2A - 1.13± 0.08 | Orb2A + Tob – 1.79 ± 0.19 | P ≤ 0.001 |
| **Orb2B stability** | | |
| Orb2B – 4.32 ± 0.53 | Orb2B + Tob – 3.95 ± 1.65 | NS |
| **Tob stability** | | |
| Tob– 1.10 ± 0.14 | Tob + Orb2A - 5.38 ± 1.76 | P=0.002 |
| Tob– 1.10 ± 0.14 | Tob + Orb2B- 6.19± 3.01 | P=0.01 |
| Tob– 1.10 ± 0.14 | Tob + Calyculin– 1.37 ± 0.22 | NS |
| Tob + Orb2A– 5.38 ± 1.76 | Tob + Orb2A +Calyculin – 2.09 ± 0.76 | P=0.002 |
| Tob + Orb2B - 6.19± 3.01 | Tob + Orb2B + Calyculin – 2.14 ± 0.11 | NS |
