# Supplementary material for: Contribution of Orb2A Stability in Regulated Amyloid-Like Oligomerization of Drosophila Orb2
Source: PLoS Biol. 2014 Feb 11;12(2):e1001786. doi: 10.1371/journal.pbio.1001786 (PMC3921104; doi:10.1371/journal.pbio.1001786)
Supplement: Text S1 — Extended materials and methods. (DOCX) [file pbio.1001786.s009.docx]

**Text S1.**

**Material and Methods:**

**Antibodies:** Anti-*Drosophila* Tob was raised against His-tagged C-terminal end of Tob in guinea pig (Pocono Rabbit Farm & Laboratory, Canadensis, PA). For all analysis both the preimmune and immune Tob serum was purified using Melon Gel IgG purification kit (Thermo Scientific). The mammalian Tob antibody was purchased from Assay Design Inc (catalogue # 905-071). All anti-Orb2 antibodies were raised against recombinant six-histidine tagged full length Orb2A protein and affinity purified against recombinant Orb2A protein. The Orb2B specific antibody was raised in rabbit against a 15 amino acid peptide KHSPSGGASGGGDAS specific to the Orb2B isoform (Sigma). The antibodies 273 were raised in rabbits (Covance) and antibody 2163, 2233 was raised in guinea pigs (Pocono). The following antibodies were obtained from hybridoma bank (Iowa) and used in the indicated dilution in the western blot anti-CSP (1:20,000), anti-Syntaxin (1:5000).

**Proteomic Analysis**: The proteomic analysis was performed using Elav::Orb2AHA flies, Elav::Orb2BHA flies, and as a control, CantonS. The analysis was performed three times each for Orb2AHA and Orb2BHA, and a total of six times for CantonS, using HA antibody to purify Orb2. Two additional experiments were performed using Orb2AHA flies and Orb2 complexes were purified using the Orb2 antibody, 2233. As a control for these experiments, identical flies were used, but guinea pig IgG was substituted. For each experiment 30 ml of adult flies were collected and decapitated by flash freezing and vortex. The heads were collected and homogenized in 20 ml lysis buffer (20 mM HEPES pH7.5, 150 mM NaCl, 2.5 mM MgCl_2_ 0.05% Igepal, 0.5% TritonX, 5% glycerol) supplemented with 10 μg/ml RNase, and 70 U/ml DNase [[1](#_ENREF_1)]. Following homogenization, the lysates were clarified two times by centrifugation at 14,000 RPM for 15 min. The lysates were incubated with 1 ml anti-HA agarose (Sigma) for 4 hrs at 4° C. Four washes were performed in lysis buffer minus MgCl_2_ and the bound proteins were eluted with 1 ml 1mg/ml HA peptide. Prior to precipitation, a methanol/chloroform extraction was performed to decrease lipid content. Precipitated pellets were solubilized in TRIS-HCl pH 8.5 and 8M Urea. TCEP (Tris(2-Carboxylethyl)-Phosphine Hydrochloride, Pierce) and CAM (Chloroacetamide, Sigma) were added to a final concentration of 5mM and 10mM, respectively. Protein suspensions were digested overnight at 37°C using Endoproteinase Lys-C at 1:50 wt/wt (Roche). Samples were brought to a final concentration of 2M urea and 2mM CaCl_2_ before performing a second overnight digestion at 37°C using Trypsin (Promega) at 1:100 wt/wt. Formic acid (5% final) was added to stop the reactions. Samples were loaded on split-triple-phase fused-silica micro-capillary columns (McDonald, et al. 2002) and placed in-line with linear ion trap mass spectrometers (LTQ, ThermoScientific), coupled with quaternary Agilent 1100 HPLCs. Fully automated 10-step chromatography run (for a total of 20 hours) was carried out for each sample, as described in[[2](#_ENREF_2)], enabling dynamic exclusion for 120 sec. The MS/MS datasets were searched using SEQUEST [[3](#_ENREF_3)] against a database of 37466 sequences, consisting of 18556 *D. melanogaster* non-redundant proteins (downloaded from NCBI on 2012-03-08), 177 usual contaminants (such as human keratins, IgGs, and proteolytic enzymes), and, to estimate false discovery rates, 18733 randomized amino acid sequences derived from each non-redundant protein entry. Peptide/spectrum matches were sorted, selected and compared using DTASelect/CONTRAST[[4](#_ENREF_4)]. Combining all runs, proteins had to be detected by at least 2 peptides, leading to FDRs at the protein and spectral levels of 0.63 and 0.15, respectively. To estimate relative protein levels, Normalized Spectral Abundance Factors (dNSAFs) were calculated for each detected protein distributing shared spectral counts based on unique peptides, as described in (Zhang, Wen, et al. 2010). PLGEM was used to calculate signal-to-noise (STN) ratios between samples and controls and derive p-values for significant enrichment of proteins in the immunoprecipitates [[5](#_ENREF_5)].

**Recombinant Proteins and *in vitro* Pull-down Assay**: Recombinant MBP-tagged Tob proteins were purified from *E. coli* using a two-step purification process using NiNta resin (Qiagen) first, eluted and dialyzed overnight in followed by purification with amylose resin (NEB), both according to the manufacturer’s protocol. Maltose binding protein was purified in a single step process with the amylose resin. Orb2A and Orb2B were *in vitro* transcribed and translated (TnT, Promega) in the presence of ^35^S-Met. OrbA or Orb2B were incubated with amylose bound Tob in 1% Igepal buffer for 2 hrs at 4° C. Unbound proteins were removed by 4 washes in binding buffer and the bound proteins were examined by SDS/PAGE followed by autoradiography. Prior to drying, the gel was stained with coomassie to ensure equal loading of the proteins. The input levels of Orb2A and Orb2B were examined by running 2 μl of the TnT reaction alongside the pulldowns.

**Phospho-Tag Staining:** Phospho tag is a dinuclear metal complex (1,3-bis[bis(pyridin-2-ylmethyl) amino]propan-2-olato dizinc(II) complex) that binds selectively to phosphate group [[6](#_ENREF_6)]. The phosphorylated Orb2 was detected using phos-tag^TM^ Biotin (Wako Pure Chemical Industries, LTD; Biotin BTL-104) using manufacturer’s protocol. In brief, Orb2 was immunoprecipitated from either adult fly head or transfected S2 cells in presence or absence of phosphatase inhibitors (Calbiochem, Phosphatase inhibitor cocktail I). To remove phosphate groups the immunoprecipitates were incubated with 10 units of calf intestinal alkaline phosphatase (New England bio lab) at room temperature for 30 minutes. The proteins were analyzed in a 4-12% gradient SDS-polyacrylamide gel (Invitrogen) and transferred into PVDF membrane. The membrane was blocked in TBS buffer containing 0.01% Tween-20 (TBS-T) for 1 hour in room temperature. The phos-tag solution was prepared by adding 5 ul of 10mmol/L Phos-tag BTL, 20ul of 10mmol/L Zn(NO_3_)_2_ , 1ul of Streptavidin conjugated Horse Redox Peroxidase (Cell Signaling) in 470 ul of TBS-T. The resulting solution was incubated for 30 min at room temperature, centrifuged at room temperature for 10 min at 14,000xg through miliporefilter YM-30. The remaining solution (the flow through was discarded) was diluted to 30 ml with TBS-T and added to the blocked PVDF membrane. After 2-4 hours the membrane was washed 3 times with TBS-T and developed using ECL reagents (PIECE, Western Dura^TM^). The same blot was subsequently Western blotted for Orb2.

**References:**

1. Tan L, Chang JS, Costa A, Schedl P (2001) An autoregulatory feedback loop directs the localized expression of the Drosophila CPEB protein Orb in the developing oocyte. Development 128: 1159-1169.

2. Florens L, Carozza MJ, Swanson SK, Fournier M, Coleman MK, et al. (2006) Analyzing chromatin remodeling complexes using shotgun proteomics and normalized spectral abundance factors. Methods 40: 303-311.

3. Link AJ, Eng J, Schieltz DM, Carmack E, Mize GJ, et al. (1999) Direct analysis of protein complexes using mass spectrometry. Nat Biotechnol 17: 676-682.

4. Tabb DL, McDonald WH, Yates JR, 3rd (2002) DTASelect and Contrast: tools for assembling and comparing protein identifications from shotgun proteomics. J Proteome Res 1: 21-26.

5. Pavelka N, Fournier ML, Swanson SK, Pelizzola M, Ricciardi-Castagnoli P, et al. (2008) Statistical similarities between transcriptomics and quantitative shotgun proteomics data. Mol Cell Proteomics 7: 631-644.

6. Kinoshita E, Takahashi M, Takeda H, Shiro M, Koike T (2004) Recognition of phosphate monoester dianion by an alkoxide-bridged dinuclear zinc(II) complex. Dalton Trans: 1189-1193.
